# Early occurrence of caffeoylshikimic acid esterase (CSE) activity in the hornwort *Anthoceros agrestis*

**DOI:** 10.1007/s00425-025-04915-7

**Published:** 2026-01-06

**Authors:** Janik Marks, Maike Petersen

**Affiliations:** Institut für Pharmazeutische Biologie und Biotechnologie, Philipps-Universität Marburg, Robert-Koch-Str. 4, 35037 Marburg, Germany

**Keywords:** Caffeoylshikimate esterase, Hornworts (Anthocerotophyta), Hydroxycinnamoyl esters and amides, *Mesotaenium endlicherianum* (Zygnematophyceae), Monoacylglycerol lipase (MAGL), Monolignol formation

## Abstract

**Main conclusion:**

The hornwort Anthoceros agrestis harbors a caffeoylshikimate esterase (CSE) displaying esterase activities with various caffeoyl- and 4-coumaroyl esters as well as lipase activity with the surrogate substrate 4-nitrophenyl butyrate.

**Abstract:**

Caffeoylshikimic acid esterase (CSE) is an enzyme of the monoacylglycerol lipase (MAGL) family shown to be involved in monolignol biosynthesis and thus has importance for lignification. To date, active CSEs have only been found in seed plants. A protein (AaCSE1) from the hornwort *Anthoceros agrestis* with 51.5% identity to the respective CSE sequence from *Arabidopsis thaliana* (AtCSE, At1g52760) displayed esterase activity with caffeoyl-5-*O*-shikimic acid as well as its 3-*O*- and 4-*O*-regioisomers. Chlorogenic acid (caffeoyl-5-*O*-quinic acid) as well as 4-coumaroyl esters were accepted with a lower affinity and catalytic efficiency. Slight activity could also be demonstrated for cleavage of the amide bond in *N*-(caffeoyl)-5-hydroxyanthranilic acid. Although AaCSE displays CSE activity and has high affinity for its substrate, this enzyme has a lower catalytic efficiency (~ 21-fold lower) compared to the CSE from *Arabidopsis thaliana*. Assays with the surrogate lipase substrate 4-nitrophenyl butyrate showed lipase activity. Thus, AaCSE1 could serve a dual function as esterase and lipase. Three putative CSE sequences from *Mesotaenium endlicherianum*, a model organism from the Zygnematophyceae, were also amplified and heterologously expressed. Only MeMAGL3 was active as a lipase. Our study showed for the first time an active CSE from a non-seed plant with dual activity as esterase/amidase as well as lipase, which could indicate a transitional state towards the evolution of more specialized CSEs predominantly involved in monolignol formation.

**Supplementary Information:**

The online version contains supplementary material available at 10.1007/s00425-025-04915-7.

## Introduction

Many important plant natural products are based on phenylpropanoid derivatives, among them lignin as a cell wall-strengthening compound and many specialized products (flavonoids, isoflavonoids, some essential oil components, stilbenes, lignans, hydroxycinnamic acid esters and amides, etc.). The aromatic amino acids l-phenylalanine and—especially in monocotyledonous plants—l-tyrosine are important precursors for the formation of these types of specialized metabolites by the phenylpropanoid pathway. In this central pathway, l-phenylalanine is deaminated to *t*-cinnamic acid by phenylalanine ammonia-lyase, and then *t*-cinnamic acid is hydroxylated by cinnamic acid 4-hydroxylase to form 4-coumaric acid. In the last step, 4-coumaric acid is activated as coenzyme A (CoA) thioester by 4-coumaric acid CoA-ligase. 4-Coumaroyl-CoA can then be either used in further pathways leading to, e.g., flavonoids or hydroxycinnamic acid esters, or reduced to 4-coumaryl alcohol as one of the building blocks of lignin (H-unit) by cinnamoyl-CoA reductase and cinnamyl alcohol dehydrogenase. The other important monolignols are coniferyl alcohol (G-unit) and sinapyl alcohol (S-units) with more elaborated substitution patterns at their aromatic rings (Boerjan et al. 2003).

How are these substitution patterns introduced? This question has long been discussed and investigated, and several pathways have been proposed and shown to exist. At least three different ways to introduce additional hydroxyl (and later methoxy) groups into 4-coumaric acid/4-coumaroyl-CoA have been described.

The most accepted way to date has been described by Vanholme et al. (2013) who showed that the activity of a caffeoylshikimic acid esterase (CSE) in *Arabidopsis thaliana* is essential for lignification. CSE releases caffeic acid from caffeoylshikimic acid, which is then further methylated to ferulic acid by caffeic acid *O*-methyltransferase. Sinapic acid is formed by 5-hydroxylation (ferulic acid 5-hydroxylase) and *O*-methylation by caffeic acid *O*-methyltransferase followed by activation as CoA-thioester by 4-coumaric acid CoA-ligase. The methylation reactions can also take place with caffeoyl-CoA, feruloyl-CoA, and/or 5-hydroxyferuloyl-CoA as substrates by caffeoyl-CoA *O*-methyltransferase. As explained above, caffeoyl-, feruloyl-, and sinapoyl-CoA can then be reduced to the respective alcohols, the monolignols, as building blocks for lignin (Boerjan et al. 2003).

The synthesis of G- and S-monolignols has been associated with the coupling of 4-coumaric acid from 4-coumaroyl-CoA with shikimic acid by a hydroxycinnamoyltransferase (HCT), namely 4-coumaroyl-CoA:shikimic acid hydroxycinnamoyltransferase (HST), followed by *meta*-hydroxylation by CYP98 to caffeoylshikimic acid to afford the caffeic acid substitution pattern (Schoch et al. 2001; Hoffmann et al. 2003). HST can also catalyze the reverse reaction and thus release caffeoyl-CoA (Hoffmann et al. 2003). This reaction, however, is rather inefficient and requires a high concentration of coenzyme A.

A direct hydroxylation of 4-coumaric acid to caffeic acid by a cytosolic 4-coumaric acid 3-hydroxylase/ascorbic acid peroxidase has been shown by Barros et al. (2019) in *Arabidopsis thaliana* and *Brachypodium distachyon*. Knockout mutants of *B. distachyon* showed more severe effects on lignin accumulation than in *A. thaliana*. Since the genome of *B. distachyon* has no orthologs for CSE, the above-mentioned pathway might be essential in this species (Saleme et al. 2017).

Caffeoylshikimic acid esterase (At1g52760) has been first described by Vanholme et al. (2013) in *A. thaliana* as an essential enzyme of lignin biosynthesis with esterase activity. CSE mutants accumulated the CSE substrate caffeoylshikimic acid and showed enhanced incorporation of 4*-*hydroxyphenyl units (H-units) into lignin. The authors could also show the cleavage of caffeoylshikimic acid to caffeic acid and shikimic acid, while caffeoyl-CoA was not formed (as in the reverse reaction of HST). Thus, the activation as CoA-thioester had to be catalyzed by a CoA-ligase afterwards. The rather similar compound chlorogenic acid [= caffeoyl-5-*O*-quinic acid; nomenclature according to Abrankó and Clifford (2017)] did not serve as a substrate for CSE (Escamilla-Treviño et al. 2014). CSE was also shown to be involved in lignification in gymnosperms such as *Larix kaempferi* (Wang et al. 2019) and other angiosperms, e.g., poplar (Wang et al. 2021). Besides being one of the key players in the formation of monolignols and thus lignin (Vanholme et al. 2013; Saleme et al. 2017; de Vries et al. 2021a), CSE is also involved in the formation of phenolic specialized products with caffeic, ferulic, and sinapic acid substitution patterns, e.g., floral volatile compounds (Kim et al. 2023), curcuminoids (Utomo et al. 2024), or montbretin A (Kruse et al. 2024).

An enzyme predominantly hydrolyzing chlorogenic acid, but also caffeoylshikimic acid derivatives, was identified from a gram-negative bacterium isolated from mycorrhizal tomato roots. The hydroxycinnamic acid esters served as the only carbon source for the growth of these bacteria (Negrel et al. 2016). Protein purification suggested that the enzyme acts as a monomeric enzyme with a molecular mass around 61 kDa and thus is much larger than known plants’ CSEs (36–37 kDa) and probably not (closely) related.

CSE belongs to the monoacylglycerol lipase (MAGL) protein family. Monoacylglycerol lipases (EC 3.1.1.23) catalyze the last step of triacylglycerol hydrolysis, releasing the last fatty acid and glycerol. A MAGL from peanut (*Arachis hypogaea*) was shown to have three active centers, one for hydrolysis of monoacylglycerols, a second lipase center for hydrolysis of lysophosphatidylcholine, and a third center responsible for acyltransferase reactions (Vijayaraj et al. 2012). In *A. thaliana*, 16 putative MAGLs could be identified in the genome, out of which 11 showed MAGL activity (Kim et al. 2016). The different MAGLs were localized in various compartments of the cell. One of them, AtMAGL3 (At1g52760), was initially described as an enzyme with acyltransferase as well as acyl hydrolase activities (Vijayaraj et al. 2012). However, in assays performed by Kim et al. (2016), a hydrolase activity was not detectable. Expression of this gene within the plant was ubiquitous, with very high levels in roots and stems. Later, AtMAGL3 was shown to be the CSE identified by Vanholme et al. (2013) as described above.

The lack of CSE genes and the respective esterase activities in model grasses (Ha et al. 2016) shows that tracheophytes not necessarily have CSEs in their phenolic metabolism. However, a bryophyte such as *Physcomitrium patens* has been reported to have convincing orthologous sequences (Renault et al. 2017; de Vries et al. 2021b), although their catalytic properties have not been elucidated. Since CSEs and MAGLs share conserved sequence motifs, the reaction catalyzed by the encoded proteins cannot be deduced from their sequences. Phylogenetic studies suggest that an ancestor of CSE might have already been present in the common ancestor of land plants (de Vries et al. 2021b). Land plants most likely have evolved from an ancestor from the Zygnematophyceae, freshwater streptophytic algae. During this conquest of land, horizontal gene transfer from soil bacteria has played an important role in achieving drought resistance (Cheng et al. 2019). One of the newly acquired genes has been shown to be phenylalanine ammonia-lyase from the phenylpropanoid pathway (Emiliani et al. 2009).

Hornworts belong to a taxon that is regarded to be quite close to the water–land transition threshold. The hornwort *Anthoceros agrestis* is known for its elaborate phenolic metabolism. Besides hydroxycinnamic acid esters of dihydroxyphenyl ethanols, lignans such as (hydroxy) megacerotonic acid and a phenolic alkaloid (anthocerotonic acid), rosmarinic acid (RA) and its 3’–O-β-d-glucoside have been found (Takeda et al. 1990; Trennheuser 1992; Trennheuser et al. 1994; Vogelsang et al. 2006). Several enzymes of the general phenylpropanoid pathway, the biosynthesis of RA and the formation of hydroxycinnamic acid esters/amides have recently been investigated in this species (Petersen 2003; Wohl and Petersen 2020a, 2020b; Busch and Petersen 2021; Busch 2022; Ernst et al. 2022; Pezeshki et al. 2022). Accordingly, additional enzymes in the biosynthesis of phenolic compounds in this organism are of interest. Among these is CSE, an enzyme involved in the establishment of diverse substitution patterns in hydroxycinnamic acid moieties, that also occur in compounds identified in *Anthoceros agrestis*.

We here describe the identification and characterization of an active CSE from the hornwort *Anthoceros agrestis*, which has – besides CSE activities – also lipase activity with the surrogate substrate 4-nitrophenyl butyrate (4-NPB). We furthermore investigated orthologous sequences from *Meso-taenium endlicherianum* to find out whether MAGL-like proteins in this member of the Zygnematophyceae already have CSE activities.

## Materials and methods

### Plant material and chemicals

A suspension culture of *Anthoceros agrestis* Paton was cultivated in CB-medium as described previously (Petersen 2003). The original callus culture had been established by Binding and Mordhorst (1991). *Mesotaenium endlicherianum* c. nägeli (SAG 12.97) was obtained as a stab culture from the “Sammlung von Algenkulturen”, Universität Göttingen (Germany). Although Busch and Hess (2022) proposed to reclassify *Mesotaenium endlicherianum* into a new genus, this study will continue to use the established taxonomy. Suspension cells were cultured on agar plates and in suspension in Bold’s Basal Medium with vitamins (BBM + V) according to SAG medium recipe no. 26 (https://www.uni-goettingen.de/de/186449.html). For propagation, 2.4–4.0 ml of a 2-week-old *Mesotaenium endlicherianum* suspension was transferred into fresh medium. Suspension cultures of *A. agrestis* and *M. endlicherianum* (Fig. S1) were shaken continuously at 100 rpm. All cultures were cultivated under continuous light (~ 15 µmol photons m^−2^ s^−1^; LI-COR Li-250A Light Meter). The sources for compounds used as substrates for CSE reactions are provided in Table S1.

### RNA isolation and cDNA synthesis

7-day-old suspension cells of *Anthoceros agrestis* were ground to a fine powder in liquid nitrogen and 50 mg transferred into 2 ml reaction tubes. Total RNA extraction was performed following the Chomczynski and Sacchi (1987) method. RNA integrity was checked by agarose gel electrophoresis; RNA was quantified photometrically. cDNA was synthesized using the RevertAid First Strand cDNA Synthesis kit (Thermo Fisher Scientific) following the manufacturer’s protocol.

### Identification and amplification of CSE and MAGL sequences

To identify putative CSE or MAGL sequences in *Anthoceros agrestis*, a BLAST search was performed with the *Arabidopsis thaliana* CSE (Vanholme et al. 2013; UniProt Q9C942; At1g52760) as bait in the hornwort database of the University of Zurich (www.hornworts.uzh.ch) (Li et al. 2020). Only sequences up to an e-value of ≤ 1.0e-50 were considered (Table S2), and the most similar homolog was identified and selected for further investigation. The BLAST search for homologous sequences in *Mesotaenium endlicherianum* was performed in the 1KP database (https://db.cngb.org/onekp/; sample code WDCW) with the AaCSE1 sequence as the query, and the same e-value threshold (≤ 1.0*e*-50) was applied (Table S3). Three putative CSE sequences were chosen for further analysis.

After designing specific primers with added restriction sites (NdeI and XhoI) (Table S4), PCR was performed under the conditions described in Table S5 to amplify the sequences from cDNA. After agarose gel electrophoresis, the amplicons were excised, purified (NucleoSpin Gel and PCR clean-up kit, Macherey–Nagel), and ligated first into pDRIVE (Qiagen) for replication in *E. coli* EZ and sequencing, and subsequently into pET-15b for expression in *E. coli* SoluBL21 (Amsbio, Abingdon, UK). Prior to expression, the sequence was verified again by sequencing (Microsynth Seqlab, Göttingen, Germany).

### Protein expression and purification

For heterologous expression, *E. coli* SoluBL21 harboring the respective expression plasmid was cultivated first overnight in LB-medium containing ampicillin (100 µg/ml) at 37 °C, 220 rpm, followed by inoculation of 100 ml TB-medium with ampicillin (for composition of media see Lessard 2013) with 1 ml of the overnight culture. The cell suspension was incubated at 37 °C and 220 rpm until an optical density at 600 nm of 0.4. Protein formation was induced by the addition of 1 mM isopropyl-β-d-1-thiogalactopyranoside and incubation at 25 °C at 180 rpm overnight. The cells were harvested by centrifugation at 6000 g (10 min, 4 °C), the pellet frozen in liquid nitrogen and then thawed on ice. The cells were resuspended in 4 ml 0.1 M potassium phosphate (KPi) buffer (pH 8.0) per gram of pellet and then incubated with about 20 mg lysozyme for 30 min on ice. The suspension was subjected to ultrasonication (four times 30 s with intermediate cooling on ice for 30 s). Cell residues were spun down at 10,000 g for 10 min at 4 °C to achieve the crude protein extract.

The crude extract was further purified by metal chelate chromatography on Ni–NTA agarose using the *N*-terminally attached 6xHis-tag. 1 ml Roti^®^Garose-His/Ni beads (Carl Roth) was pipetted into a column and equilibrated with 10 ml binding buffer (50 mM KPi pH 8.0, 10 mM imidazole, 300 mM NaCl). The crude extract was adjusted to 10 mM imidazole and 300 mM NaCl for binding, applied to the column, and incubated for 1 h on ice under gentle agitation. The resulting flow-through, washing, and elution fractions were collected for SDS-PAGE analysis. Washing steps were performed with three times 2 ml wash buffer (50 mM KPi pH 8.0, 20 mM imidazole, 300 mM NaCl) and elution with three times 1 ml elution buffer (50 mM KPi pH 8.0, 50 mM imidazole, 300 mM NaCl). The elution fractions were desalted by PD10-columns (GE Healthcare) and transferred into 0.1 M KPi buffer pH 7.0. The protein concentration was determined according to Bradford (1976). The purified protein was stored at -80 °C until further use.

### SDS-PAGE and Western blot analysis

To verify the expression of the proteins of interest, samples from the purification process (see above) were separated by SDS-PAGE (Laemmli 1970) and stained with Coomassie Brilliant Blue R250 or subsequently transferred onto a PVDF membrane by semi-dry Western blotting as described by Mahmood and Yang (2012) using the Towbin et al. (1979) buffer system. The detection was performed with the primary mouse anti-6xHis-tag monoclonal antibody (Thermo Fisher Scientific; MA1-21,315) and the secondary goat anti-mouse antibody conjugated to alkaline phosphatase (Life Technologies; A16087) and a color reaction using 5-brom-4-chloro-3-indoxyl-phosphate and nitro blue tetrazolium chloride (https://www.sysy.com/protocols/westernblot-ap-detection).

### Determination of kinetic data for esterase activities

To assess substrate acceptance and determine specific catalytic parameters, the enzymes were tested in assays containing either caffeoyl or 4-coumaroyl esters/amides (see Table S1) as substrates under the conditions described in Table S6. The reaction was terminated by the addition of 20 µl 6 M HCl, followed by two extractions with 300 µl ethyl acetate each. The collected organic phases were evaporated by vacuum centrifugation, and the residue redissolved in 100 µl 40% methanol (v/v) acidified with 0.01% H_3_PO_4_ (v/v). The samples were either analyzed by HPLC and/or LC–MS. Control reactions were measured with proteins purified from *E. coli* harboring pET-15b without inserted DNA. For kinetic measurements, linear turnover was assured at the lowest and highest substrate concentrations. The biochemical and kinetic parameters were determined with multiple technical replicates (*n* = 3–9).

### Photometric lipase assays

Lipase activity was measured using a double-beam spectrophotometer (Specord 200 Plus; Analytik Jena) in quartz cuvettes. A constant temperature was achieved by a Peltier-tempered cuvette holder. The non-native surrogate lipase substrate, 4-nitrophenyl butyrate (4-NPB), was used in the activity tests. The assay conditions for kinetic analyses are shown in Table S7. 4-NPB was added immediately before use and mixed thoroughly so that no substrate droplets were detectable. This solution was stored on ice to ensure as little autohydrolysis of 4-NPB as possible. The cold solution was pipetted into quartz cuvettes and incubated at 25 °C for 3 min before starting the reaction with 50 µl of the enzyme solution. The resulting 4-nitrophenolate ion (ε = 18,000 M^−1^ cm^−1^) was detected at 405 nm (McCain and Zhang 2002). Controls were measured with proteins purified from *E. coli* harbouring pET-15b without inserted DNA.

### LC–MS analysis

A HPLC 1260 system (Agilent Technologies) equipped with a Multospher 120 RP18 column (250 × 2 mm, 5 µm particle size, CS-Chromatographie Service) was used for analysis. The mobile phase consisted of 0.1% (v/v) aqueous formic acid (A) and 0.1% (v/v) formic acid in acetonitrile (B). Measurements were carried out using either the long or the short gradient method, each with distinct temperature and flow rate settings: long method – 20 °C, flow rate 0.25 ml/min, 0–40 min 5% B → 100% B; 40–45 min 100% B; 45–45.1 min 100% → 5% B; 45.1–55 min 5% B; short method – 25 °C, flow rate 0.50 ml/min, 0–10 min 5% B → 100% B; 10–15 min 100% B; 15–15.1 min 100% → 5% B; 15.1–20 min 5% B. Detection was performed with a diode array detector and a micrOTOF-Q III mass spectro-meter with electrospray ionization (ESI) source (Bruker Daltonics). For the analysis of the hydroxycinnamic moieties released by CSE, 10 µl were injected and analyzed in the negative ionization mode.

### Ethanolic extracts of *Anthoceros agrestis* suspension cells

Plant material of the *Anthoceros agrestis* suspension cultures was frozen and freeze-dried. 20 mg finely ground dry plant material was transferred into 2 ml reaction tubes and extracted with 2 ml 70% ethanol by 10 min ultrasonication at 70 °C. The supernatant was decanted after centrifugation at 3000 g for 10 min and diluted 1:10 with 50% MeOH/0.01% H_3_PO_4_. To remove suspended particles, the reaction tubes were centrifuged again at 16,000 g for 10 min, and the extracts analyzed by LC–MS with the long gradient method in the negative ionization mode (see above).

#### Bioinformatic analysis

The phylogenetic analysis of 64 (putative) CSE and MAGL sequences (Table S8) was performed using MEGA11 with the Maximum Likelihood algorithm (1000 bootstraps) and the JTT matrix-based model. The sequences were collected by BLAST search with AaCSE1 as query in the 1KP database (https://db.cngb.org/onekp/), Phytozome14 (https://phytozome-next.jgi.doe.gov/), and the University of Zurich hornwort database (https://www.hornworts.uzh.ch/en.html) or were taken from published data. Modelling was performed with AlphaFold3 (https://alphafoldserver.com) and the alignments visualized with BIOVIA Discovery Studio Visualizer (https://www.3ds.com/products/biovia/discovery-studio/visualization).

## Results

### Identification of potential CSE or MAGL sequences in *Anthoceros agrestis* and *Mesotaenium endlicherianum*

To identify homologous sequences in *Anthoceros agrestis,* the previously characterized CSE from *A. thaliana* (Vanholme et al. 2013; UniProt Q9C942; At1g52760) was used as the query sequence. The BLASTp search was conducted utilizing the hornwort database hosted by the University of Zurich (www.hornworts.uzh.ch) (Li et al. 2020), targeting the *Anthoceros agrestis* Bonn genome. A total of eight potential sequences in the genome were identified up to an e-value of 1.0e–50 (Table S2), and the sequence with the best e-value was selected for biochemical analysis (Sc2ySwM_117.2740.1 = AaCSE1). A successful PCR was performed using cDNA as a template to confirm the presence of the gene and its expression in the used suspension cultures. Sequencing revealed a single nucleotide difference in comparison to the scaffold at base 415, resulting in an amino acid substitution from l-methionine to l-leucine at position 139 (M139L). The exchange was found to be adjacent to the putative catalytic serine; however, the three putative essential amino acids GxSxG (Wang et al. 2021) in the conserved domain were not affected.

To identify potential MAGL or CSE sequences in *Mesotaenium endlicherianum*, AaCSE1 was used as a query template in the 1KP database (https://db.cngb.org/onekp/; sample code WDCW). Here, a total of four sequences with an e-value of ≤ 1.0e-50 were found (Table S3), of which three were more closely examined (WDCW_scaffold_2007872 = MeMAGL-like1; WDCW_scaffold_2047059 = MeMAGL-like2; WDCW_scaffold_2008809 = MeMAGL3). A PCR was performed to amplify the sequences.

For heterologous protein formation, all identified sequences were ligated into the expression vector pET-15b after sequence verification and introduced into *E. coli* SoluBL21. Prior to conducting enzyme assays, SDS-PAGE and Western blots were performed to confirm the expression of the desired enzyme (Figs. S2 and S3). All samples showed bands at the expected size (including the *N*-terminally attached 6xHis-tag). Co-purified proteins seen on the SDS-PAGE gels also occur in empty vector controls (Fig. S3).

### Molecular and biochemical analysis of putative CSE/MAGL sequences from *Anthoceros agrestis* and *Mesotaenium endlicherianum*

AaCSE1’s (GenBank PV962689) open reading frame includes 972 nucleotides encoding a protein of 324 amino acid residues with a molecular mass of 35.87 kDa without the attached 6xHis-tag and 38.04 kDa including it. The amino acid sequence displayed an identity of 51.5% (EMBOSS Needle) to the respective sequence from *A. thaliana* (At1g52760; Vanholme et al. 2013) (Table S9). AaCSE1 catalysed the hydrolysis of the ester bond in caffeoyl-5-*O*-shikimic acid and its regioisomers, namely caffeoyl-4-*O*-shikimic acid and caffeoyl-3-*O*-shikimic acid. Furthermore, it was able to catalyze the hydrolysis of caffeoyl-5-*O*-quinic acid (chlorogenic acid) and its regioisomers (3-*O*- and 4-*O*-) to a lesser extent. Very low activity was observed with 4-coumaroyl-5-*O*-quinic acid. RA was not hydrolysed. Remarkably, *N*-(caffeoyl)-5-hydroxyanthranilic acid was also accepted as a substrate, albeit only in small traces. This shows the ability of AaCSE1 to also hydrolyze amide bonds. All esterase activities were proven by LC–MS (Fig. S4). Lipase activity for AaCSE1 was observed using 4-NPB as a substrate (see below).

The open reading frame of MeMAGL-like1 (GenBank PV962726) comprises 1014 base pairs, encoding a protein of 338 amino acids. The calculated molecular mass of the protein is 37.41/39.58 kDa without and with 6xHis-tag. No introns were identified in the genomic sequence. Esterase and lipase assays were performed with caffeoyl-5-*O*-shikimic acid, caffeoyl-5-*O*-quinic acid, RA, and 4-NPB, but no activity was observed.

The open reading frame of MeMAGL-like2 (GenBank PV962727) was not interrupted by introns and comprises 1134 base pairs, encoding a protein of 378 amino acids. The calculated molecular mass of the protein is 42.23/44.40 kDa without and with the 6xHis-tag. A transmembrane domain was detected using DeepTMHMM 1.0 (Hallgren et al. 2022) (Fig. S5), which accounts for the size difference between this enzyme and the other isoforms. Enzyme assays were performed with the same four substrates tested previously for MeMAGL-like1; however, no activity was detectable.

MeMAGL3’s (GenBank PV962700) genomic sequence did not include introns. The open reading frame consisted of 1032 base pairs translating into 344 amino acids, resulting in a molecular mass of 39.19/37.02 kDa with and without 6xHis-tag. Esterase assays with the aforementioned hydroxy-cinnamic acid esters did not result in detectable activity. However, lipase assays with 4-NPB were successfully conducted (see below).

## Kinetic characterization of AaCSE1

AaCSE1 showed a high promiscuity with respect to the esters and amides that were hydrolysed. It efficiently hydrolysed ester bonds in various hydroxycinnamoyl esters with a preference for esters with caffeoyl moieties (caffeoyl-5/4/3-*O*-shikimic acids, caffeoyl-5/4/3-*O*-quinic acids). Remarkably, 4*-*coumaroyl esters and the amide *N*-(caffeoyl)-5-hydroxyanthranilic acid were also hydrolysed. In addition, AaCSE1 showed lipase activity with the lipase substrate 4-NPB with the highest catalytic efficiency (Table 1).

**Table 1:** Kinetic parameters for AaCSE1 and MeMAGL3. k_cat_ was calculated with the molecular mass of 38.04 kDa (including the 6x-His-tag) for AaCSE1 and 39.19 kDa (including the 6x-His-tag for MeMAGL3. The parameters are presented including standard deviation (SD) where appropriate

Substrate	K_m_ [µM]	V_max_ [µkat kg^−1^]	k_cat_ [s^−1^]	k_cat_/K_m_ [M^−1^ s^−1^]
**AaCSE1**				
caffeoyl-5-*O*-shikimic acid (*n* = 6 ± SD)	36.4 ± 7.4	174.3 ± 13.6	0.0066 ± 0.0005	181.9 ± 22.5
caffeoyl-5-*O*-shikimic acid (ECB-method) (*n* = 6)	30.6	163.1	0.0062	202.9
caffeoyl-5-*O*-quinic acid (chlorogenic acid) (*n* = 9 ± SD)	425.1 ± 107.4	416.8 ± 23.6	0.0159 ± 0.0009	37.3 ± 12.5
4-nitrophenyl butyrate (4-NPB) (*n* = 3)	662.1	13,760.0	0.5233	790.4
**MeMAGL3**				
4-nitrophenyl butyrate (4-NPB) (*n* = 12 ± SD)	1560 ± 331	293,400 ± 27,014	11.5 ± 1.1	7357 ± 1159

**Fig. 1:**
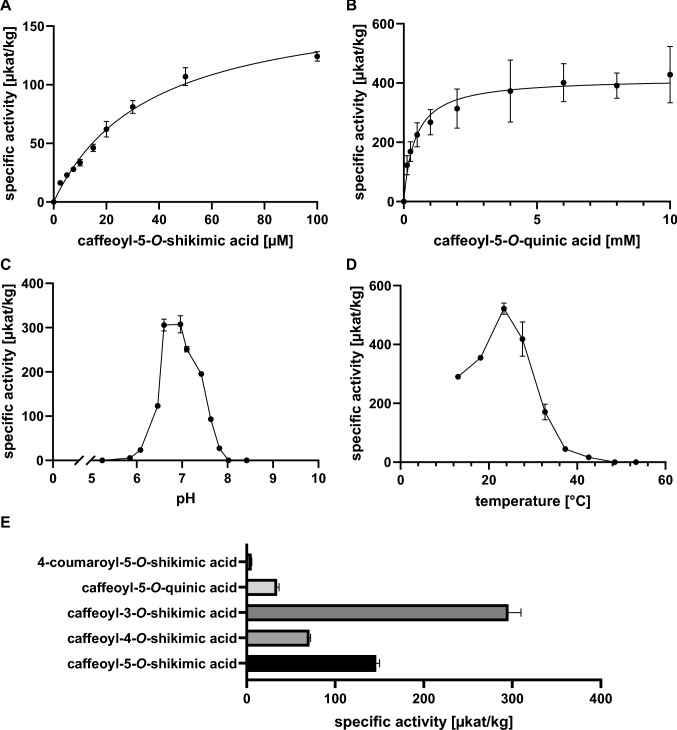
Esterase activity of AaCSE1. **A** Substrate saturation curve for caffeoyl-5-*O*-shikimic acid (*n* = 6 ± SD). **B** Substrate saturation curve for caffeoyl-5-*O*-quinic acid (chlorogenic acid) (*n* = 9 ± SD). **C** pH optimum (*n* = 3 ± SD). **D** Temperature optimum (*n* = 3 ± SD). E Relative activity of AaCSE1 with different hydroxycinnamoyl esters (50 µM) (*n* = 3 ± SD)

The highest affinity was found for caffeoyl-5-*O*-shikimic acid (K_m_ 36.44 µM; determined by Michaelis–Menten plots; Fig. 1A, Table 1) or 30.56 µM (determined by the Eisenthal–Cornish–Bowden method (Cornish-Bowden and Eisenthal 1978; Fig. S6). The latter method was employed additionally, as substrate saturation was not fully achieved in the assays. Caffeoyl-5-*O*-quinic acid (chlorogenic acid) was also accepted by the enzyme; however, it exhibited an approximately tenfold lower affinity (K_m_ 425.10 µM; Fig. 1 B; Table 1). Using caffeoyl-5-*O*-quinic acid, the temperature optimum and the pH-optimum of AaCSE1 were determined to be at 23.4 °C and pH 6.60–6.96, respectively (Fig. 1C, D).

**Fig. 2:**
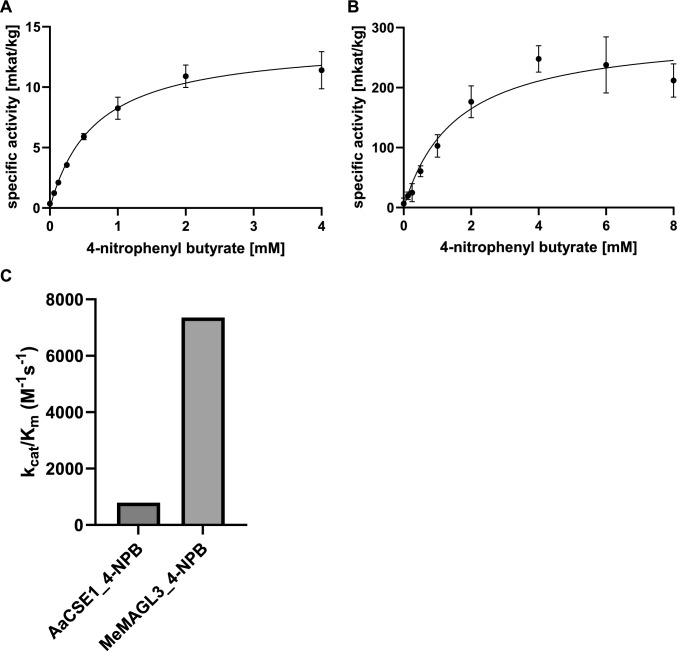
Michaelis–Menten kinetics for lipase activities of AaCSE1 and MeMAGL3 using 4-nitrophenyl butyrate (4-NPB) as substrate. **A** Substrate saturation curve for AaCSE1 (*n* = 3 ± SD). **B** Substrate saturation curve for MeMAGL3 (*n* = 12 ± SD). **C** Comparison of the catalytic efficiencies of AaCSE1 and MeMAGL3

The surrogate substrate 4-NPB to test lipase activity was accepted with a low affinity (662.10 µM) but with a high reaction velocity (13,760.0 µkat kg^−1^; Fig. 2A, Table 1). Compared to the hydroxycinnamoyl esters, the reaction was approximately 80 times faster than the hydrolysis of caffeoyl-5-*O*-shikimic acid (174.3 µkat kg^−1^) and 30 times faster compared to caffeoyl-5-*O*-quinic acid (416.8 µkat kg^−1^). Overall, the catalytic efficiency (k_cat_/K_m_) with the lipase substrate was about three times higher compared to the best esterase substrates.

A direct comparison between the regioisomers of caffeoylshikimic acid, 4-coumaroyl-5-*O*-shikimic acid, and caffeoyl-5-*O*-quinic acid (chlorogenic acid) at a fixed substrate concentration of 50 µM confirmed the preference for esters with caffeoyl moieties compared to 4-coumaroyl moieties (Fig. 1 E). This observation implies that AaCSE1 may function as a true CSE, rather than as a promiscuous esterase that also hydrolyses 4-coumaroylshikimic acid to a similar degree. Interestingly, the preferred substrate under the selected conditions was caffeoyl-3-*O*-shikimic acid. However, no evidence for the presence of caffeoyl-3-*O*-shikimic acid was found in ethanolic extracts of *Anthoceros agrestis* suspension cultures. Solely the 4-*O*- and 5-*O*-esters could be identified by retention times and mass spectra matching to authentic standards (Fig. S7).

### Verification of esterase products by LC–MS

To verify the esterase activity with hydroxycinnamoyl esters and amides, LC–MS analysis was conducted for the reaction products caffeic acid and 4-coumaric acid. Caffeic acid and 4-coumaric acid were detected at *m/z* 179.03 [M-H]^–^ and 163.04 [M-H]^–^, respectively. The acceptance was shown for different caffeoyl and 4-coumaroyl esters and one amide: caffeoyl-5-*O*-, -4-*O*- and -3-*O*-shikimic acids and caffeoyl-5-*O*- and -4-*O*-quinic acids with a high to medium turnover and 4-coumaroyl-5-*O*-shikimic acid, 4-coumaroyl-5-*O*-quinic acid, caffeoyl-3-*O*-quinic acid and *N*-(caffeoyl)-5-hydroxyanthranilic acid to a considerably lower extent (Fig. S4, Table S1). No activity was detectable with 4-coumaroyl-3-*O*- and -4-*O*-quinic acids (probably due to a restricted detection limit) and rosmarinic acid.

### Kinetic characterization of AaMAGL3

MeMAGL3 was tested with hydroxycinnamoyl esters as well as with 4-NPB. Hydrolysis could only be observed for the lipase substrate 4-NPB, showing that MeMAGL3 is not a CSE. The affinity for 4-NPB was rather low (K_m_ 1560 µM) (Table 1), but the reaction velocity was high. Substrate saturation curves showed a decline at higher substrate concentrations (Fig. 2), either caused by real substrate inhibition or by the low solubility of the substrate, or both. Thus, only apparent kinetic parameters were determined. The catalytic efficiency of MeMAGL3 as a lipase with 7357 M^−1^ s^−1^ exceeds the lipase efficiency of AaCSE1 (790 M^−1^ s^−1^) at least tenfold.

### Bioinformatic analysis

Pairwise global sequence alignments of the four analyzed sequences together with AtCSE (At1g52760) were performed utilizing the EMBOSS Needle tool to compare sequence identity and similarity between the enzymes. Comprehensive alignment results are provided in Table S9. The identity ranged from 30.4% to 51.5%, with the highest identity level between AtCSE and AaCSE1, and the similarity ranged from 44.8% to 68.6%.

AaCSE1 and MeMAGL3, the two enzymes confirmed to possess enzymatic activity, were aligned with previously characterized CSE enzymes from other species (Fig. S8). Only enzymes with verified hydrolytic activity towards one or more caffeoylshikimic acid regioisomers (except MeMAGL3) were included in the alignment. The aim of the analysis was to assess the integrity of conserved motifs, vital for enzyme activity and substrate specificity in CSEs. It revealed the presence of both conserved hydrolase motifs (C2, C3; GxSxG) in AaCSE1 as well as MeMAGL3 (Vijayaraj et al. 2012; Wang et al. 2021). The acyltransferase motif (C1; HxxxxD) is conserved as well. The conserved MAGL motif according to de Vries et al. (2021b) only partially shows the postulated typical exchange in CSEs of two hydrophobic amino acid residues (L–L/V/I) to two hydrophilic ones (F–S) as seen in AtCSE. In AaCSE1, these amino acids are L–S. MeMAGL3 presents the typical MAGL motif (L–L) at this position.

**Fig. 3:**
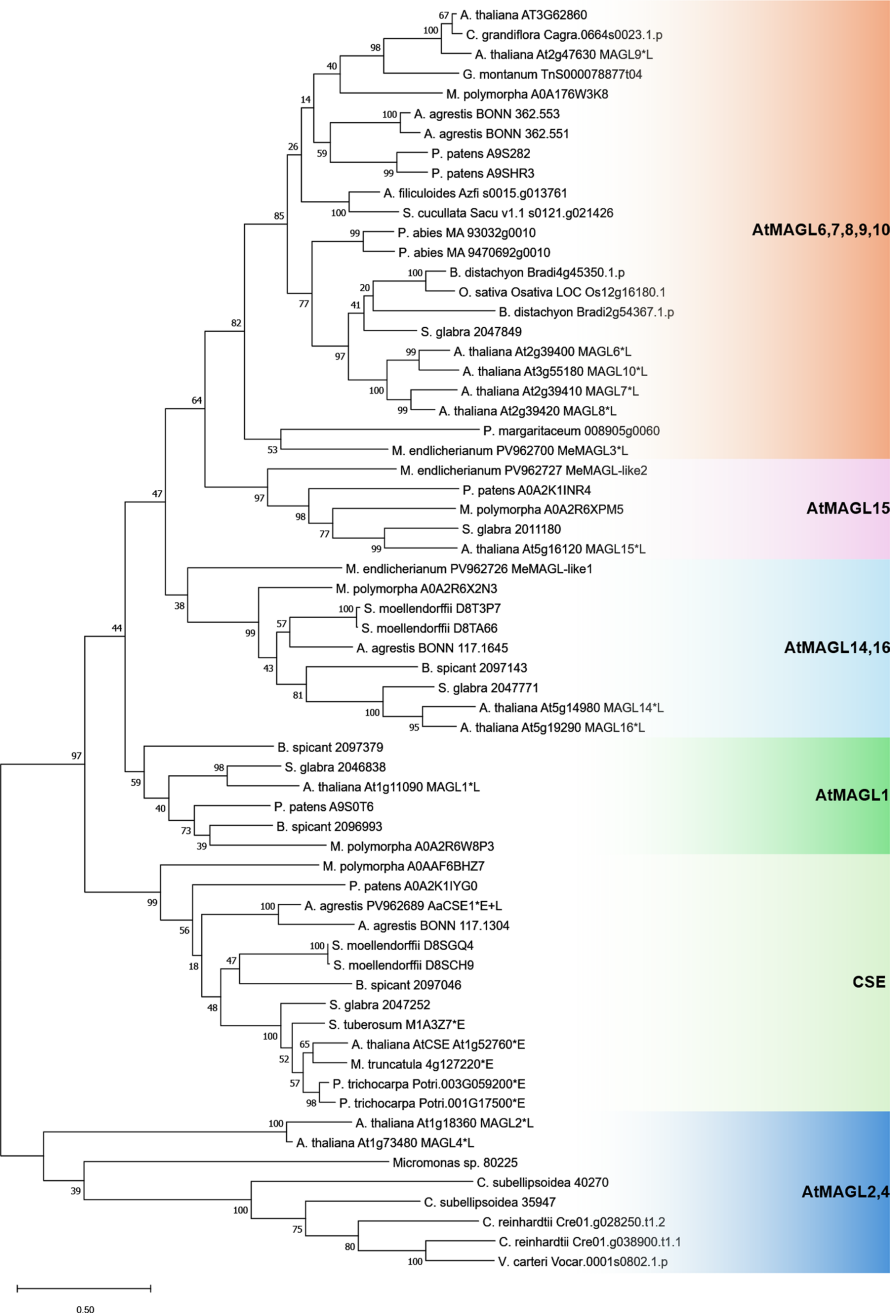
Phylogenetic tree constructed with the MEGA 11 Maximum Likelihood algorithm (1000 bootstraps) of 64 putative and confirmed CSE and MAGL amino acid sequences (for detailed information see Table S8), 18 with verified catalytic activity (*), 6 with reported esterase activity (E) with caffeoylshikimic acid, 13 with proven lipase activity (L)

Phylogenetic analysis was conducted using a selection of (putative) CSE sequences from a diverse range of plant species that encompass green algae to spermatophyta. Whenever possible, full-length sequences were chosen to ensure the accuracy of the phylogenetic tree. Of the 64 CSE/MAGL sequences used to calculate the tree, six CSEs with confirmed esterase (E) and 13 MAGLs with verified lipase (L) activity were marked. Notably, all enzymes accepting caffeoyl-5-*O*-shikimic acid clustered within a distinct branch of the tree, the CSE clade, that also includes AtCSE = AtMAGL3 (At1g52760) (Vanholme et al. 2013; Kim et al. 2016). One sequence each from the other included bryophytes, *Physcomitrium patens* and *Marchantia polymorpha*, clustered with the CSE clade, suggesting that they may also possess CSE activity. The next sequences most similar to AtCSE from *Physcomitrium patens* and *Marchantia polymorpha* grouped into the AtMAGL clades (Fig. 3) as defined by Kim et al. (2016). This is also true for additional sequences from *Anthoceros agrestis*, except for one sequence that is located on a neighboring branch adjacent to AaCSE1 and shows high sequence similarity (59.0% identity/69.6% similarity, with the highest dissimilarity due to an inserted stretch of 45 amino acid residues). The similarity between the putative PpCSE and MpCSE amino acid sequences with AaCSE1 is 63.6% and 60.1%, respectively, while the similarity to AtCSE is marginally lower at 63.0% and 57.6% (Table S10).

The *Mesotaenium endlicherianum* enzymes cluster further away from the esterase-active enzymes in separate MAGL clades. The clade including MeMAGL3 (clade AtMAGL6,7,8,9,10) encompasses other members with proven lipase activity (Fig. 3).

## Discussion

Monoacylglycerol lipases (MAGL) belong to the α/β-hydrolase protein family. Members of the MAGLs not only show hydrolase activities with mono- and diacylglycerols and lysophosphatidylcholine but have also shown acyltransferase activity, thus transferring fatty acid moieties from CoA-activated fatty acids to mono- or diacylglycerols. The acyltransferase activity is related to the HxxxxD-motif (C1), whereas two different hydrolase active centers (GxSxG; C2 and C3) were found in the amino acid chain (Vijayaraj et al. 2012; Wang et al. 2021). Whereas C1 and C3 are buried inside the protein, C2 is located at the surface (Vijayaraj et al. 2012). Astonishingly, members of this family have been characterized as CSE involved in the formation of mono-lignols and other phenolic compounds with dihydroxylated or methoxylated phenylpropanoid moieties (e.g., caffeoyl, feruloyl, sinapoyl) (Vanholme et al. 2013). The importance for lignin synthesis has been proven by a number of experiments where downregulation or knockout of CSE in seed plants resulted in a decrease in lignin content and a change in lignin composition (Vanholme et al. 2013; Ha et al. 2016; Kim et al. 2016; Vargas et al. 2016; Saleme et al. 2017; Wang et al. 2019; de Vries et al. 2021a; Jang et al. 2021; Serrani-Yarce et al. 2021; Qiao et al. 2025). Moreover, the formation of other specialized metabolites, such as curcuminoids (Utomo et al. 2024), floral volatiles (Kim et al. 2023), or montbretin A (Kruse et al. 2024) is also affected by activity changes of CSE. Therefore, CSE plays a pivotal role in plant metabolism. However, some monocotyledonous plants have been reported to lack CSE sequences and activity (Ha et al. 2016; Serrani-Yarce et al. 2021). The presence of CSE/MAGL-like sequences has been shown for chloro- and streptophytic algae, but it is unknown whether they perform CSE activities (de Vries et al. 2021b).

True lignin has been reported to be present in embryo-phytes like ferns and seed plants while it is absent from bryophytes and algae, although monolignol epitopes could be labelled by antibodies (Kremer et al. 2004; Ligrone et al. 2008). Lignin and secondary walls have been described in the red alga *Calliarthron cheilosporioides* (Martone et al. 2009). This was considered a convergent evolution. A CSE-like sequence as well as sequences putatively encoding other enzymes of monolignol biosynthesis have been discovered in the transcriptome of the related species *Calliarthron tuberculosum*, although a copy of an HST, forming the substrate for CSE, was missing (Xue et al. 2022).

In hornworts such as *Anthoceros agrestis*, lignin has to date not been found. Nevertheless, hornworts are known for an elaborate phenolic metabolism, as, e.g., *Anthoceros agrestis* contains RA and other phenolic derivatives such as anthocerodiazonin (Takeda et al. 1990; Trennheuser 1992; Trennheuser et al. 1994; Vogelsang et al. 2006). Enzymes known to be involved in the biosynthesis of RA in Lamiaceae have been detected on molecular and biochemical levels in *Anthoceros agrestis* (Petersen 2003; Wohl and Petersen 2020b, 2020a; Busch and Petersen 2021) except the ester-forming enzyme (rosmarinic acid synthase) and a cytochrome P450 (CYP98) actively catalyzing the *meta*-hydroxylation of RA-precursors or 4-coumaroylshikimic acid. A hydroxycinnamoyltransferase (HCT) from *Anthoceros agrestis* (UniProtKB A0A8K1E0Z7) catalyzes the formation of 4-coumaroylshikimic acid and 4-coumaroyl-3-hydroxyanthranilic acid, but not 4-coumaroyl-4’–hydroxyphenyllactic acid, while the only CYP98 (CYP98A147; UniProtKB A0A8K0ZJB3) in *Anthoceros agrestis* accepted 4-coumaroylanthranilic acid and 4-coumaroyl-3-hydroxyanthranilic acid and only poorly 4-coumaroylshikimic acid and 4-coumaroyl-4’–hydroxyphenyllactic acid (Ernst et al. 2022). Thus, the formation of the presumed substrate for CSE, caffeoylshikimic acid, still is questionable.

Renault et al. (2017) found that the single CYP98 in the moss *Physcomitrium patens* is essential for cuticle formation and gametophore development and suggested that not 4-coumaroylshikimic acid but 4-coumaroylthreonic acid is formed by a HCT and then *meta*-hydroxylated. Alber et al. (2019) showed for the same CYP98 from *P. patens* that 4-coumaroylanthranilic acid was hydroxylated efficiently while 4-coumaroylshikimic acid was a poor substrate. Similar results were obtained for CYP98 from the lycophyte *Selaginella moellendorffii* and the fern *Pteris vittata*. Functional hydroxycinnamoyl-CoA:shikimic acid HCTs forming the substrates for CYP98 were identified in *P. patens* and *Marchantia polymorpha* (Kriegshauser et al. 2021). Sequences with convincing similarity to known CSE sequences, however, were reported to be missing in *P. patens* (Ha et al. 2016), although the analysis by de Vries et al. (2021b) showed one sequence each from *Physcomitrium patens* and *Marchantia polymorpha* in the proposed CSE clade as also found in our study (Fig. 3). For this reason, it is highly interesting that the hornwort *Anthoceros agrestis* harbors CSE-like sequences, and we were able to demonstrate that the *Anthoceros agrestis* protein encoded by the sequence most similar to the CSE of *A. thaliana* (UniProtKB Q9C942; At1g52760; Vanholme et al. 2013) exhibits CSE activity, while also retaining lipase activity. Both activities were characterized biochemically by enzyme assays utilizing various hydroxycinnamoyl esters as well as an amide as substrates for CSE activity and the surrogate substrate 4-NPB for lipase activity.

Reinvestigation of the genomes of *Physcomitrium patens* and *Marchantia polymorpha* using the AaCSE1 amino acid sequence as bait returned one sequence of each species that clustered with proven CSEs from other plants (Fig. 3). It is therefore probable that the genomes of *P. patens* and *M. polymorpha* contain a protein sequence encoding for an enzyme with CSE activity, as demonstrated for *Anthoceros agrestis*. It remains to be investigated whether these proteins also possess multiple activities, such as esterase, amidase, and lipase.

AaCSE1 hydrolyzes three regioisomers of caffeoylshikimic acid, namely caffeoyl-3-*O*-, -4-*O*-, and 5-*O*-shikimic acid, as well as the corresponding quinic acid esters. The highest relative activity was shown with caffeoyl-3-*O*-shikimic acid, which was about twice the activity with caffeoyl-5-*O*-shikimic acid. However, ethanolic extracts from *Anthoceros agrestis* suspension cultures contained caffeoyl-5-*O*-shikimic acid as the main isomer (besides high amounts of RA), while caffeoyl-4-*O*-shikimic acid was a minor constituent, and caffeoyl-3-*O*-shikimic acid was undetectable. It can therefore be assumed that AaCSE1 in vivo acts on the suitable endogenous substrate, namely caffeoyl-5-*O*-shikimic acid. In comparison, 4-coumaroyl-5-*O*-shikimic acid was only accepted with less than 4% of the activity observed for caffeoyl-5-*O*-shikimic acid.

Comparing the kinetic data of AaCSE1 with those of AtCSE (Vanholme et al. 2013) shows that the hornwort enzyme has a higher affinity towards caffeoylshikimic acid (K_m_ AaCSE1 36.4 µM, AtCSE 96.5 µM) but a far lower maximum reaction velocity (V_max_ AaCSE1 0.174 mkat/kg, AtCSE 9.3 mkat/kg), resulting in a 21-fold lower catalytic efficiency.

Three caffeoylquinic acid regioisomers were hydrolyzed by AaCSE1 as well, while a low conversion could only be shown for 4-coumaroyl-5-*O*-quinic acid. Interestingly, cleavage of the amide bond in *N*-(caffeoyl)-5-hydroxyanthranilic acid could be observed to a very low level. RA could not serve as a substrate. A certain substrate promiscuity has already been reported for CSEs from, e.g., *A. thaliana* (Vanholme et al. 2013), *Populus trichocarpa* (de Vries et al. 2021a), and *Crocosmia crocosmiifolia* (Kruse et al. 2024).

AaCSE1 also displayed lipase activity with 4-NPB with about fourfold catalytic efficiency compared to the turn-over of caffeoyl-5-*O*-shikimic acid. However, the affinity for 4-NPB was about 20 times lower than for caffeoyl-5-*O*-shikimic acid. 4-NPB, however, is not a natural substrate for lipases, which might explain the low affinity. Only one CSE from a different species, *A. thaliana*, has previously been tested for lipase activity: AtMAGL3, which later was renamed AtCSE, was tested with various lipid substrates but could not hydrolyze them (Kim et al. 2016). Unfortunately, to our knowledge, no other CSE has been tested with a lipase substrate in parallel to hydroxycinnamic acid esters. Thus, we cannot conclude whether this is a special feature of AaCSE1 or a common feature of all CSEs.

Three CSE/MAGL-like sequences were amplified from cDNA from *Mesotaenium endlicherianum*, a member of the Zygnematophyceae. Although all three proteins were expressed in transformed *E. coli*, only one protein (MeMAGL3) exhibited activity as lipase. Esterase activity was not detectable. MeMAGL3 displayed a rather low affinity for 4-NPB as a surrogate substrate, but a high reaction velocity resulting in a very efficient turnover despite the observed decline of measurable reaction velocity at higher substrate concentrations (Table 1, Fig. 1).

Kim et al. (2016) analyzed the MAGL family in *A. thaliana* and identified 16 members, out of which 11 showed lipase activity. The enzymes showed differing localizations in the endoplasmic reticulum/Golgi network (among those also AtMAGL3, which corresponds to AtCSE), the cytosol, oil bodies, and plastids. Wang et al. (2019, 2021) proposed a localization of CSEs from *Larix kaempferi* and poplar in the plasma membrane and the endoplasmic reticulum; however, these sequences, as well as the CSE sequences from *A. thaliana* and *Anthoceros agrestis*, did not show transmembrane regions in analyses performed by DeepTMHMM (https://services.healthtech.dtu.dk). Our enzymatic analyses were performed with soluble and purified protein fractions, giving no indication of a membrane-bound protein.

Enzymes belonging to the MAGL-group are widespread in the plant kingdom. In their phylogenetic analysis, de Vries et al. (2021b) identified two major clades, one of them restricted to streptophytes, the other also present in chlorophytes. Both, AaCSE1 and MeMAGL3 as well as the two other sequences from *M. endlicherianum* without proven activity belong to the latter clade. To date, only this clade has been shown to contain a subclade with CSE activity centered around AtMAGL3 and also containing AaCSE1. The interesting fact is that AaCSE1 not only exhibits esterase but also lipase activities, which, to our knowledge, has previously never been shown for a CSE. Since in most cases only CSE activity has been tested (without examining lipase activity), we cannot conclude that this dual activity is a special feature of *Anthoceros agrestis* CSE. If this were the case, it could show a transition state of a MAGL enzyme from lipase to CSE, which might have taken place during evolution. The change of only one of the two amino acid residues in the MAGL motif (de Vries et al. 2021b) proposed to differ between CSEs (F–S) and MAGL lipases (L–L/V/I) in AaCSE1 (L–S) might corroborate this evolutionary step from lipases to CSEs. In *Mesotaenium endlicherianum*, as a member of the Zygnematophyceae, we have not been able to show CSE activity; only MeMAGL3 proved to be an active enzyme in our hands and catalysed the hydrolysis of 4-NPB. The above-mentioned MAGL motif is L–L in MeMAGL3 as proposed for lipases in the corresponding sequence. The sequences from *Physcomitrium patens* and *Marchantia polymorpha* sorted into the CSE clade (Fig. 3) neither show the conserved MAGL (Pp: F–A, Mp: L–S) nor the CSE signature. While these two amino acids are the same in *Marchantia polymorpha* and *Anthoceros agrestis*, the sequence in *Physcomitrium patens* is closer to the proposed CSE motif.

AlphaFold3 was employed to generate the 3D structures of the enzymes described in this study as well as the well-established AtCSE; BIOVIA Discovery Studio Visualizer was used for structural alignment. The overall structure of CSEs and MAGL(-like) proteins is rather similar (Fig. S9), revealing an almost identical orientation of the active sites as well as a lid-like structure (shown in yellow) that might limit access to the active center. The amino acid residues involved in catalysis are located in an interior cavity and seem not to be directly accessible without substantial structural fluidity. The sequence of approximately 35 amino acid residues (residues 190 to 225 in AaCSE1) may function as a flexible lid-like structure similar to the hydrophobic helix described in human MAGLs (Labar et al. 2010; Scalvini et al. 2016).

When comparing the MAGL motif (de Vries et al. 2021a, b) and the active center in AaCSE1 and AtCSE with MeMAGL3, several key differences can be observed (Fig. S10). AaCSE1 is of particular interest for this comparison due to its potentially unique dual substrate affinity for lipids and caffeoylshikimic acid. Additionally, it currently is the most divergent functional CSE regarding this binding domain. The differences between the substrate binding domains (amino acid residues numbered 1 to 12, blue box Fig. S8) of MeMAGL3 and AtCSE/AaCSE1 were narrowed down to three putative key substitutions to facilitate CSE activity: Leu5 → Ser – this change was already predicted by de Vries et al. (2021a, b). Met8 → Leu and Ser12 → Pro. They were regarded as potentially essential, mainly due to their proximity and orientation towards the active site and therefore the opportunity to directly interact with caffeoylshikimic acid. However, no definitive conclusion can currently be drawn regarding which individual amino acid residue or combination thereof contributes precisely to the substrate specificity of CSEs. The structure of AaCSE could provide us with deeper insights into clarifying this question, as AaCSE exhibits both activities despite its differences from canonic MAGLs and CSEs.

The presence of a CSE in the hornwort *Anthoceros agrestis* is an interesting new finding, since our knowledge of the enzymatic machinery of *Anthoceros* to date lacks the enzyme catalyzing the *meta*-hydroxylation of 4-coumaroylshikimic acid and thus providing the main substrate of AaCSE1, caffeoyl-5-*O*-shikimic acid or other regioisomers. However, the demonstration of esterase as well as amidase and lipase activities in a member of the MAGL family is novel. Further research will be required to assess whether the *Physcomitrium patens* and *Marchantia polymorpha* sequences that group within the CSE clade exhibit properties comparable to those of AaCSE1, and to clarify the activities and roles of CSE-like enzymes in streptophytes.

## Supplementary Information

Below is the link to the electronic supplementary material.

Supplementary file1 (PDF 2376 KB)

## Data Availability

The authors declare that the data supporting the findings of this study are available within the paper and its Supplementary Information. Further information is available from the corresponding author upon reasonable request.

## Abbreviations

4-NPB: 4-Nitrophenyl butyrate
CSE: Caffeoylshikimic acid esterase
HCT: Hydroxycinnamoyltransferase
HST: Hydroxycinnamoyl-CoA:shikimic acid hydroxycinnamoyltransferase
MAGL: Monoacylglycerol lipase
RA: Rosmarinic acid
