# Supplementary material for: Early occurrence of caffeoylshikimic acid esterase (CSE) activity in the hornwort Anthoceros agrestis
Source: Planta. 2026 Jan 6;263(2):49. doi: 10.1007/s00425-025-04915-7 (PMC12774947; doi:10.1007/s00425-025-04915-7)
Supplement: Supplementary file 1 — Supplementary file1 (PDF 2376 KB) [file 425_2025_4915_MOESM1_ESM.pdf]

Supplementary Material for

Early occurrence of caffeoylshikimic acid esterase (CSE) activity in the hornwort *Anthoceros agrestis*

Janik Marks, Maike Petersen*

Supplementary Figures

A B

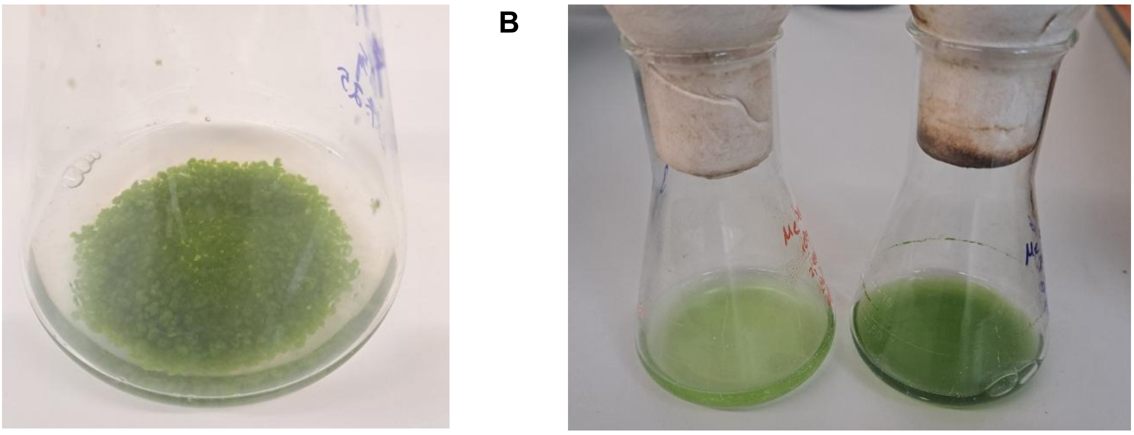

Fig. S1 A *Anthoceros agrestis* suspension culture in CB-M medium (5 days after propagation). **B** *Mesotaenium endlicherianum* suspension cultures 4 days after propagation (left), 18 days after propagation (right)

A B

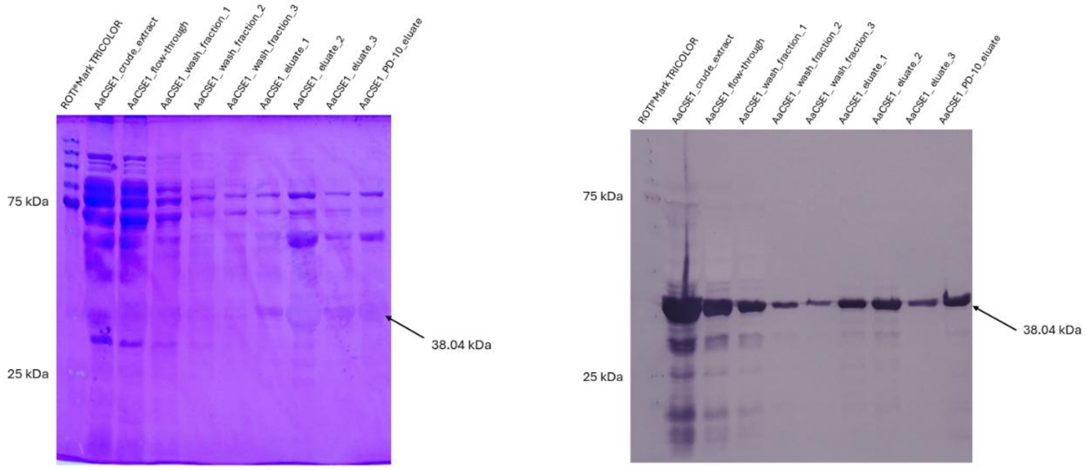

Fig. S2 SDS-PAGE (**A**) stained with Coomassie Brilliant Blue R250 and Western-blot (**B**) of AaCSE1 heterologously expressed in *E. coli* SoluBL21 and purified by NiNTA-affinity chromatography and PD-10 columns. The arrows point to the bands of AaCSE1

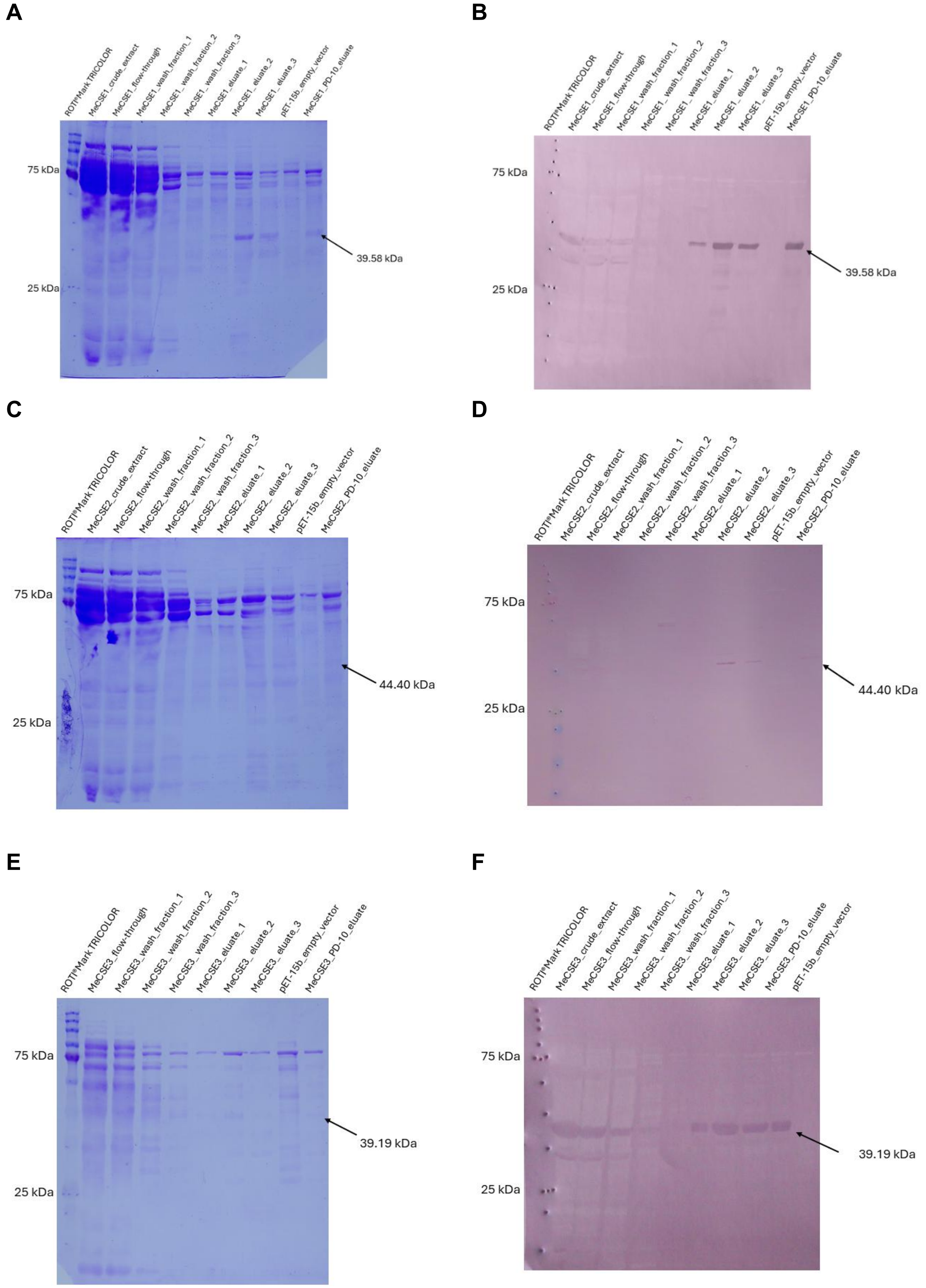

Fig. S3 SDS-PAGE (left) and Western-blot (right) of MeputCSE1 (**A**, **B**), MeputCSE2 (**C**, **D**) and MeMAGL3 (**E**, **F**) heterologously expressed in *E. coli* SoluBL21 and purified by NiNTA-affinity chromatography and PD-10 columns. Gels (**A**, **C**, **E**) were stained with Coomassie Brilliant Blue R250. The arrows point to the bands of the respective protein of interest

A caffeoyl-5-*O*-shikimic acid

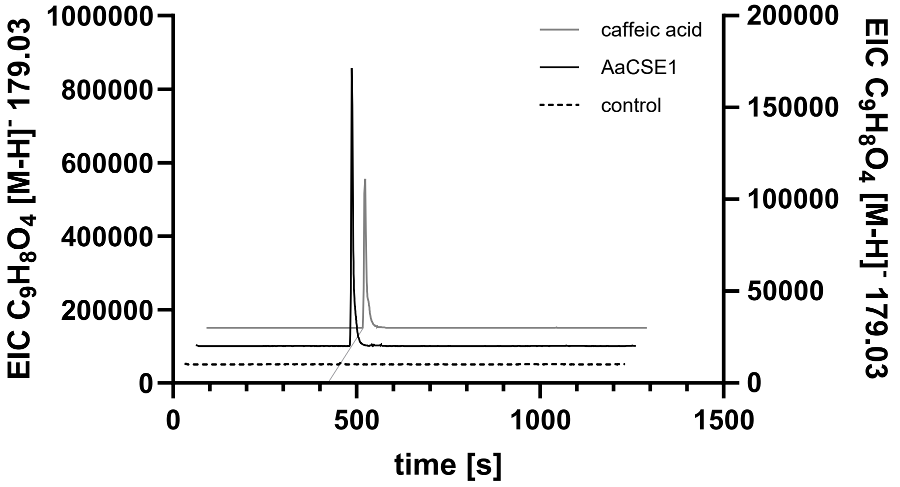

B caffeoyl-4-*O*-shikimic acid

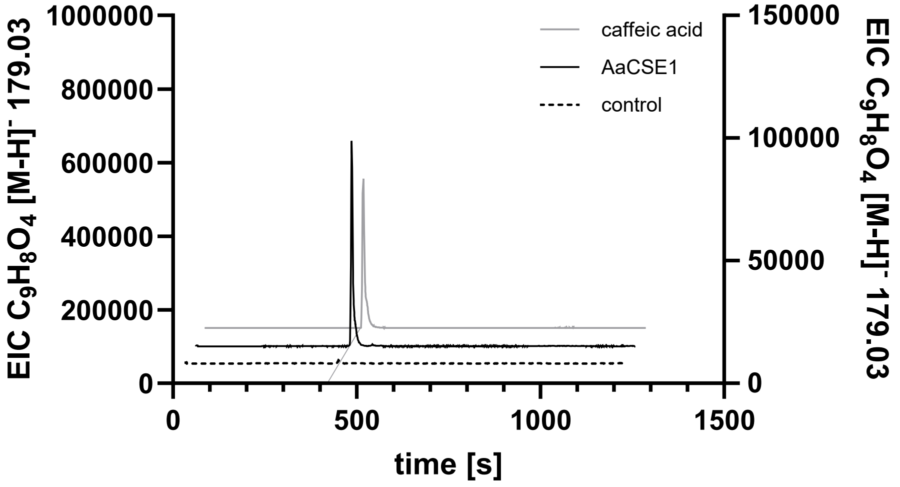

C caffeoyl-3-*O*-shikimic acid

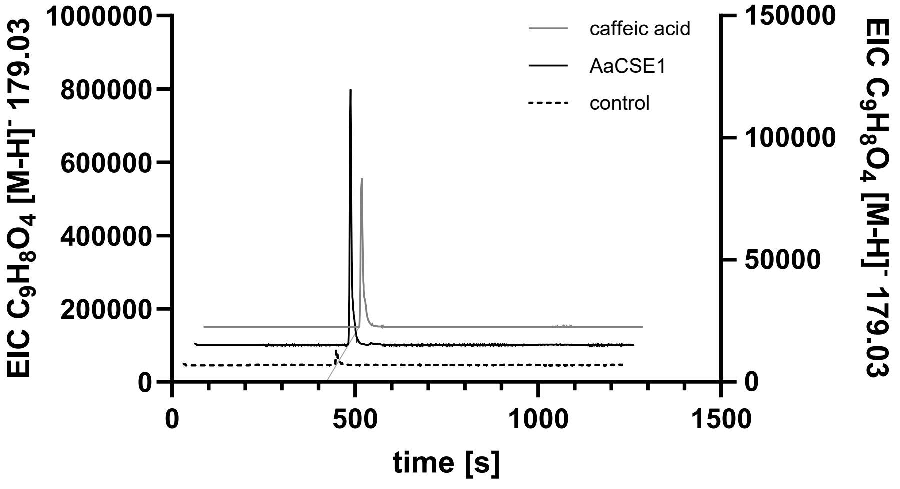

D caffeoyl-5-*O*-quinic acid

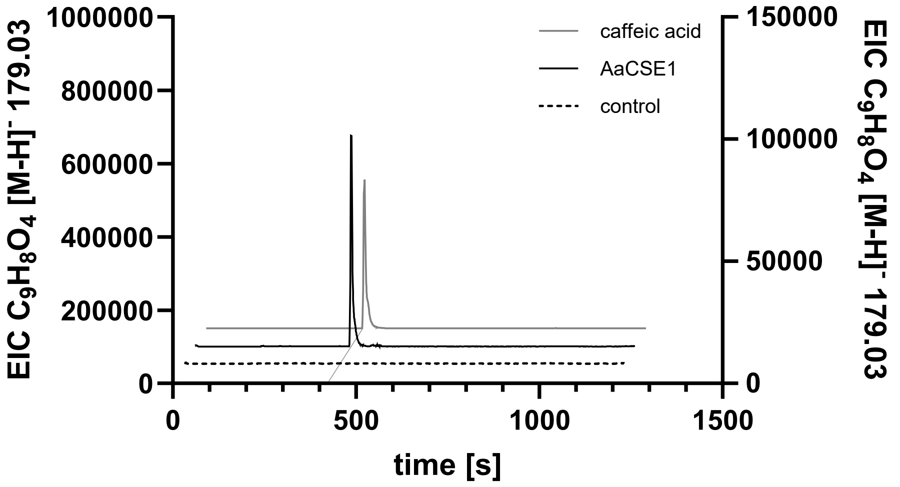

E caffeoyl-4-*O*-quinic acid

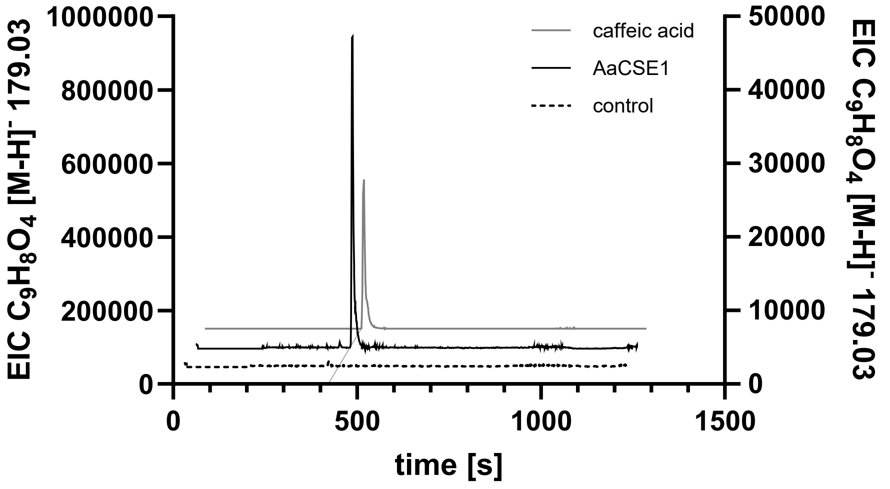

F caffeoyl-3-*O*-quinic acid

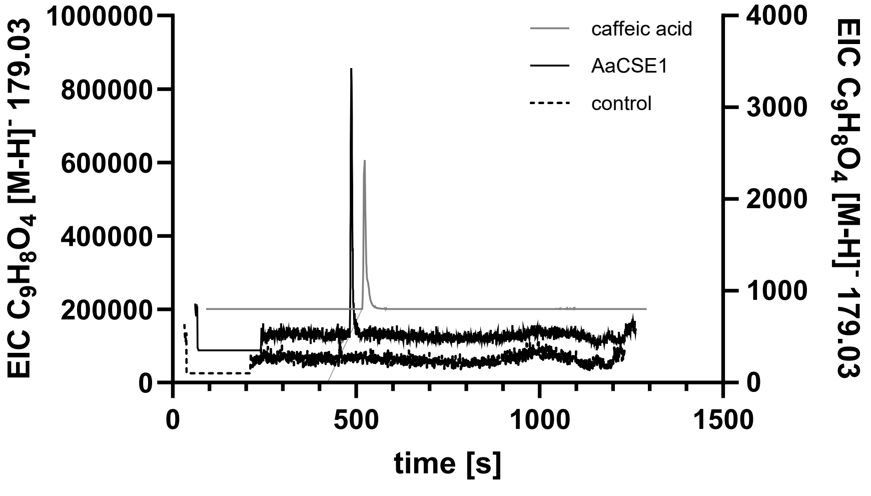

G *N*-(caffeoyl)-5-hydroxyanthranilic acid

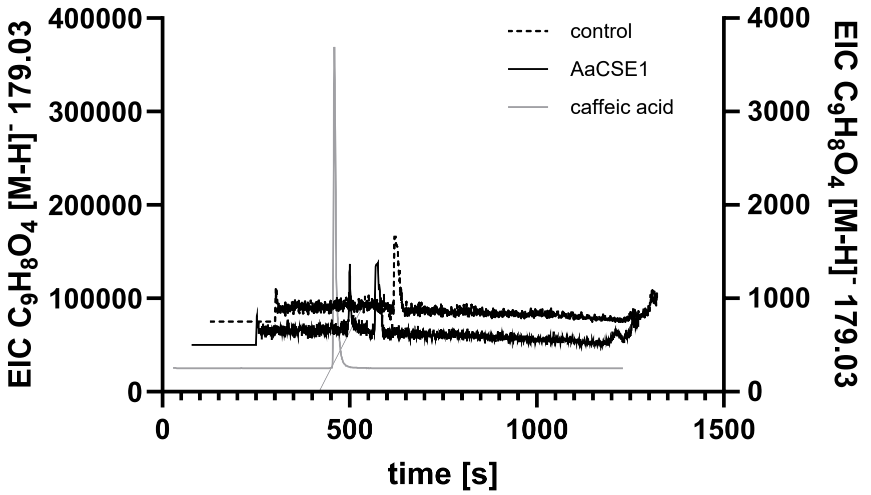

H 4-coumaroyl-5-*O*-shikimic acid

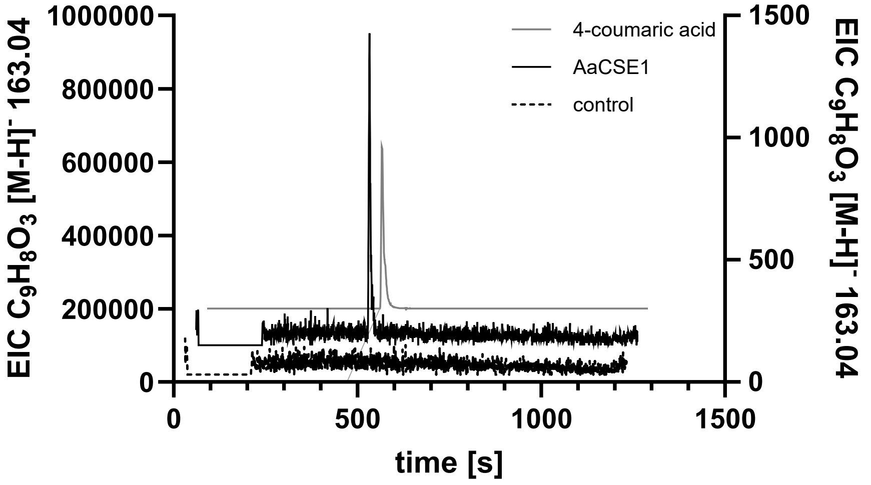

I 4-coumaroyl-5-*O*-quinic acid

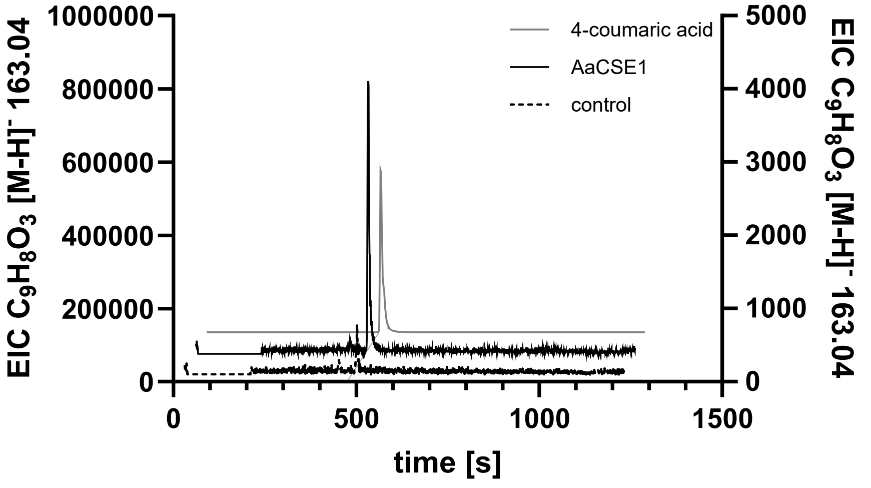

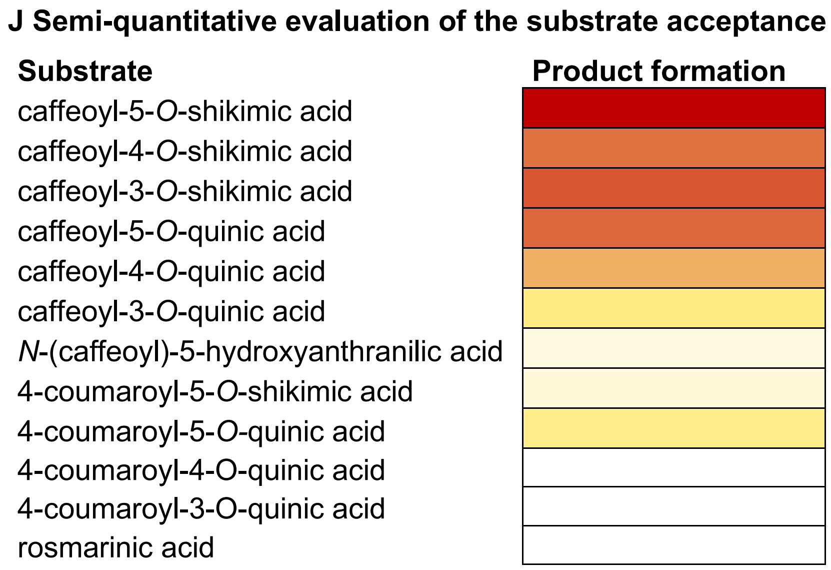

Fig. S4 Extracted ion chromatograms (EIC) of substrate acceptance assays of AaCSE1 (**A-I**) and semi-quantitative evaluation of substrate acceptance (**J**) from red (very well accepted) to green (only poorly accepted). The applied substrates were **A** caffeoyl-5-*O*-shikimic acid, **B** caffeoyl-4-*O*-shikimic acid, **C** caffeoyl-3-*O*-shikimic acid, **D** caffeoyl-5-*O*-quinic acid, **E** caffeoyl-4-*O*-quinic acid, **F** caffeoyl-3-*O*-quinic acid, **G** *N*-(caffeoyl)-5-hydroxyanthranilic acid, **H** 4-coumaroyl-5-*O*-shikimic acid, **I** 4-coumaroyl-5-*O*-quinic acid. The expected mass to charge ratio (*m/z*) as well as the molecular formula of the expected products, 4-coumaric acid or caffeic acid, are stated on the Y-axes; grey line – EIC of 25 µM standards, either caffeic ($[M-H]^-$ *m/z* 179.03) or 4-coumaric acid ($[M-H]^-$ *m/z* 163.04); black dashed line (control) – assays incubated with heat-denatured protein; continuous black line – assays under the conditions stated in Table S6. Both black lines refer to the right Y-axis, the grey line to the left Y-axis. For clear visualization of the offset of the chromatograms, a diagonal thin grey line was added. **J** Heat map showing the relative turnover of substrates by AaCSE1 from red (high turnover) to white (no turnover)

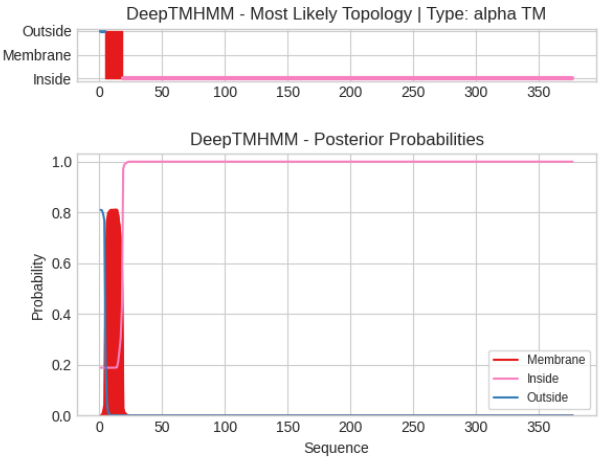

Fig. S5 MeCSE2 transmembrane domain, detected by DeepTMHMM-1.0 by DTU Health Tech (https://services.healthtech.dtu.dk/services/DeepTMHMM-1.0/)

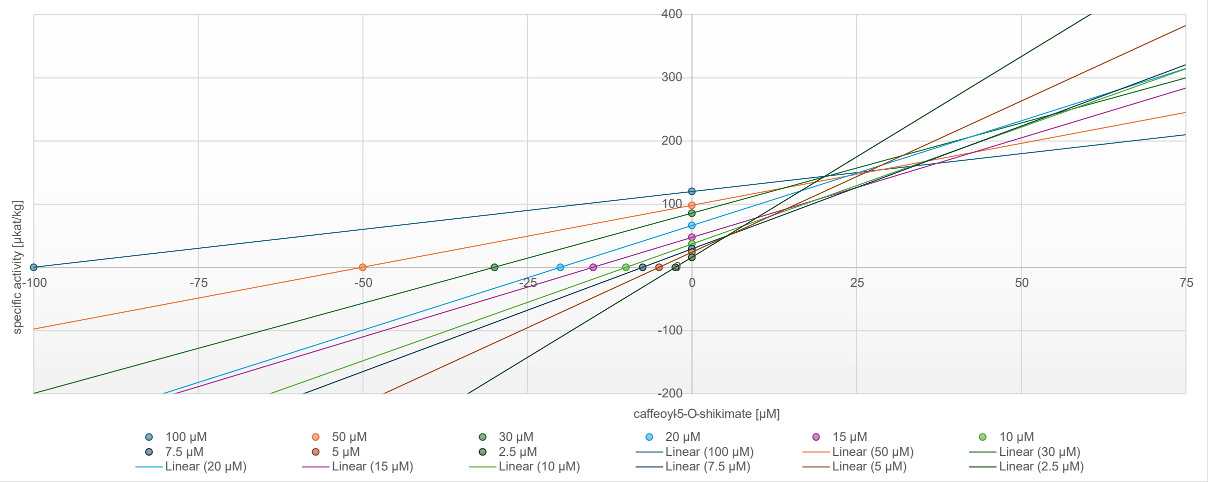

Fig. S6 Substrate saturation assays of AaCSE1 with caffeoyl-5-*O*-shikimic acid analysed with the method by Cornish-Bowden and Eisenthal (1978) to graphically estimate the kinetic parameters (K_m and V_{max})

A *Anthoceros agrestis* suspension culture (2 d) ethanolic extract

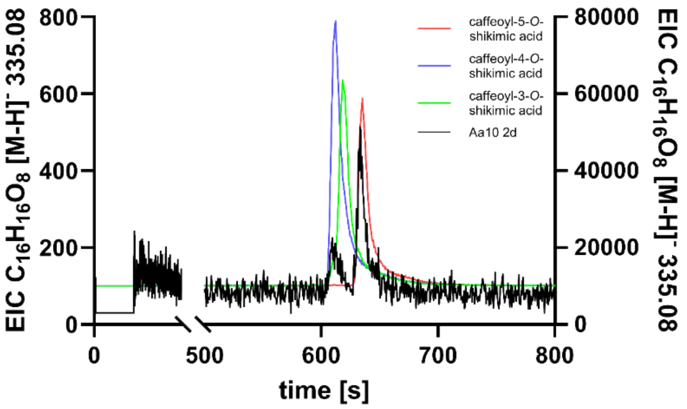

B *Anthoceros agrestis* suspension culture (7 d) ethanolic extract

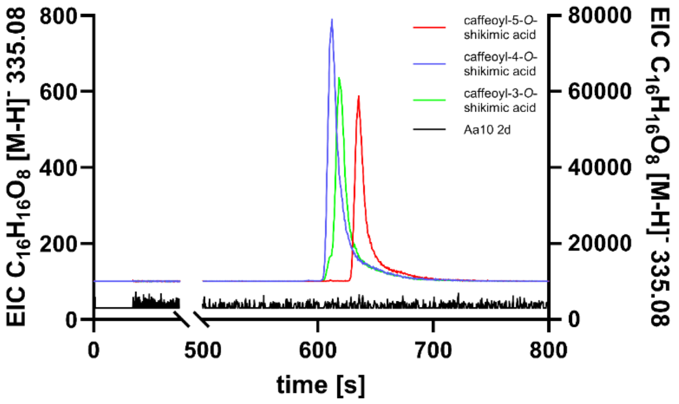

Fig. S7 Analysis of an ethanolic extract of a suspension culture of *Anthoceros agrestis*. Extracted ion chromatogram (EIC) of caffeoyl shikimic acid regioisomers ($[M-H]^-$ *m/z* 335.08). Regioisomers exhibit a slight offset in retention time. Low amounts of caffeoyl-5-*O*-shikimic acid and caffeoyl-4-*O*-shikimic acid were found 2 days after transfer into fresh CB-M medium (**A**). After 7 days no caffeoylshikimic acid esters were detected (**B**). The black line is plotted against the left Y-axis, and the standards were plotted against the right Y-axis

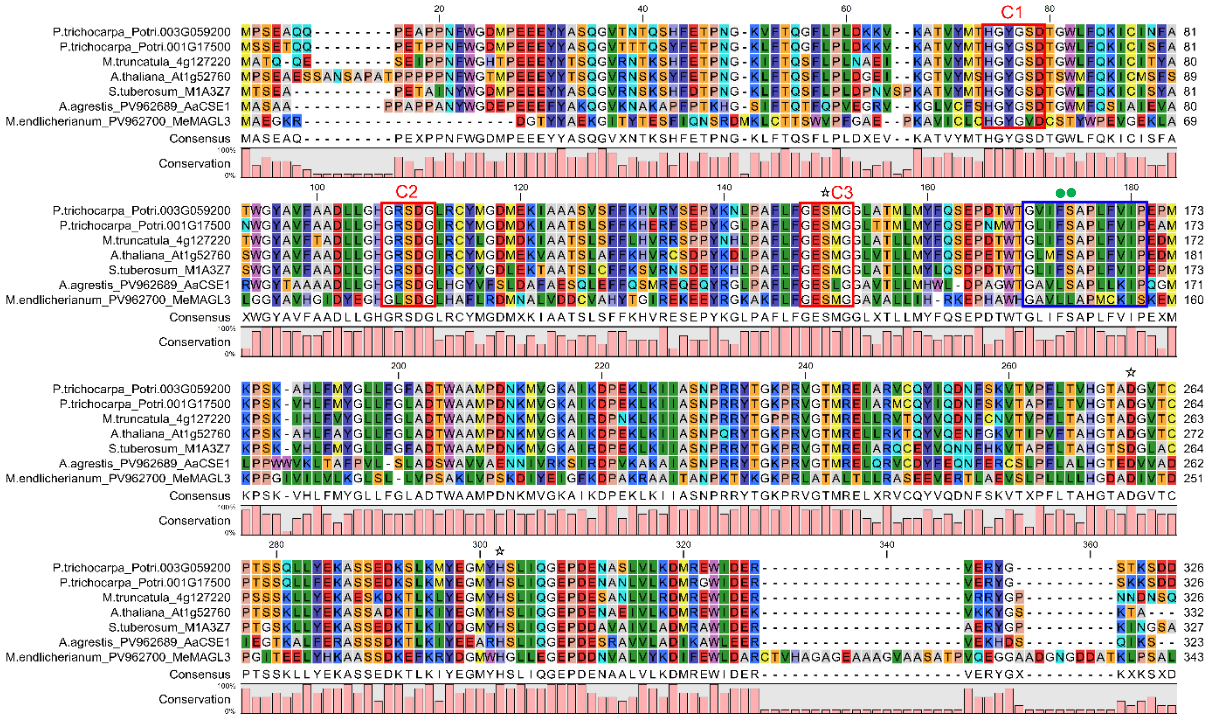

Fig. S8 Sequence alignment of active CSEs with caffeoylshikimic acid esterase activity, AaCSE1 with esterase and lipase activity and MeMAGL3 with lipase activity. Red boxes mark the HX_4D acyltransferase motif (C1) and the GXSXG hydrolase motifs (C2, C3) (Vijayaraj et al. 2012). Amino acids of the conserved catalytic triad (S, D, H) are marked with stars (☆) (Wang et al. 2019). The conserved MAGL motif according to de Vries et al. (2021) is marked with a blue box and the two amino acid residues in this motif putatively responsible for the change of MAGL (LL) to CSE (FS) activity with green dots

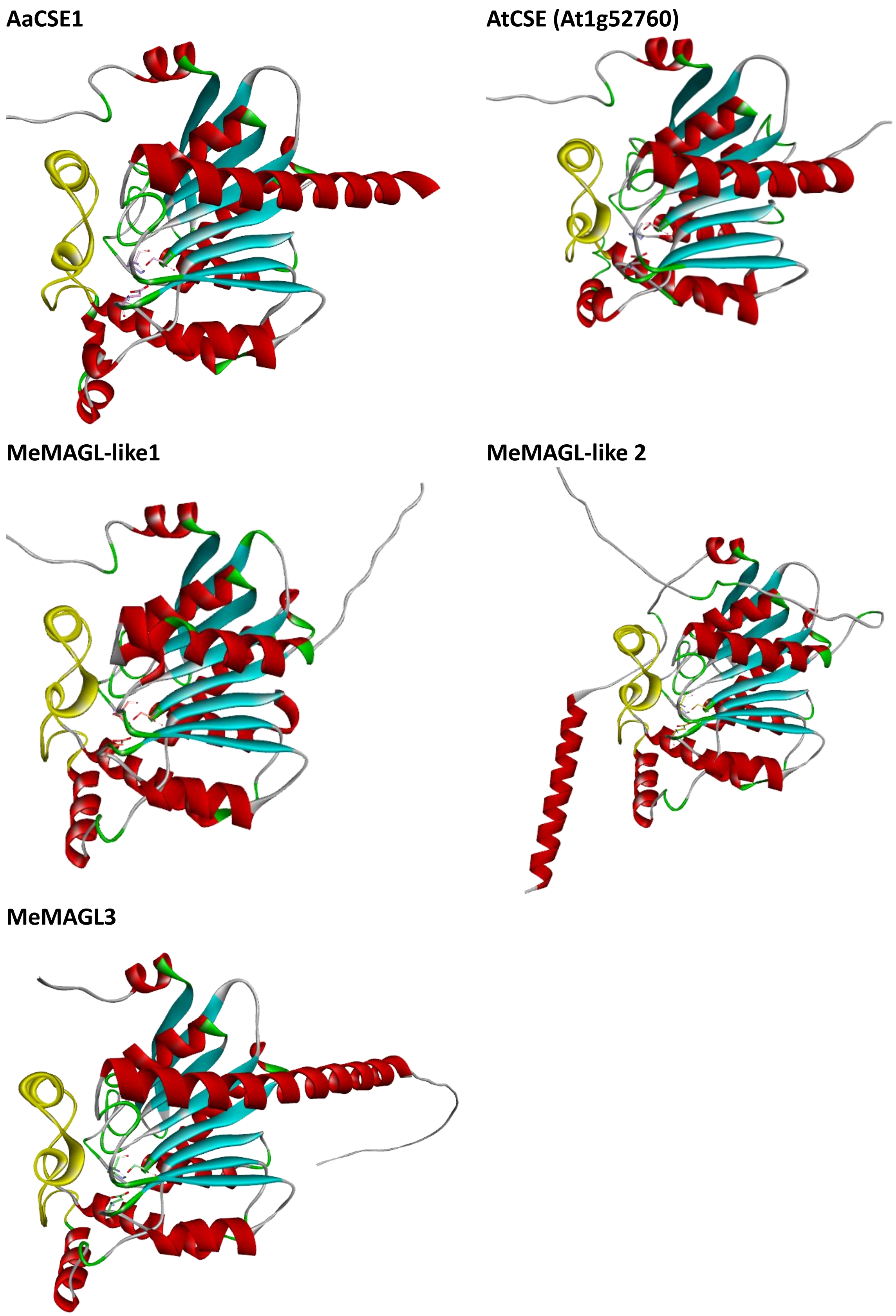

Fig. S9 Structure of CSE/MAGL proteins from *Anthoceros agrestis* (AaCSE1), *Arabidopsis thaliana* (AtCSE) and *Mesotaenium endlicherianum* (MeMAGL3). 3D models generated by AlphaFold3 and visualization in BIOVIA Discovery Studio Visualizer. The catalytic site shown as stick model, which is covered by putative lid-like structure (yellow)

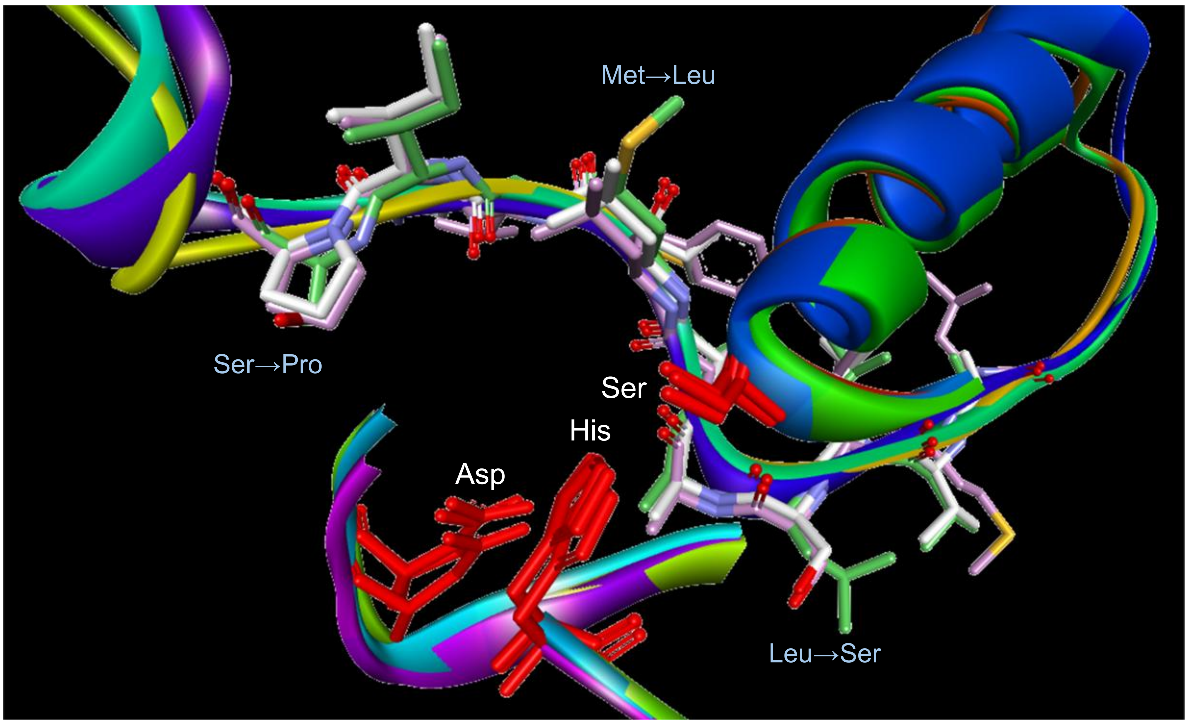

Fig. S10 Alignment of the active center of AaCSE1 (grey), AtCSE (pink) and MeMAGL3 (green) proteins from *Anthoceros agrestis*, *Arabidopsis thaliana* and *Mesotaenium endlicherianum*. 3D models generated by AlphaFold3 and visualization in BIOVIA Discovery Studio Visualizer. The amino acids in red represent the catalytic triad (serine, histidine and aspartic acid). The 12 amino acid residues of the MAGL motif are shown in stick representation, important changes between MAGL and CSE are indicated (light blue)

Supplementary Tables

Table S1 Substrates and acceptance tested in AaCSE1 esterase acceptance assays

Substrate	Structure	Acceptance
caffeoyl-5-*O*-shikimic acid<br>(CAS 73263-62-4), Benchchem	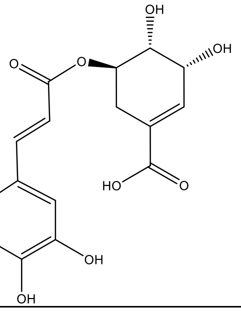	positive
caffeoyl-4-*O*-shikimic acid<br>(CAS 180842-65-3), Akos	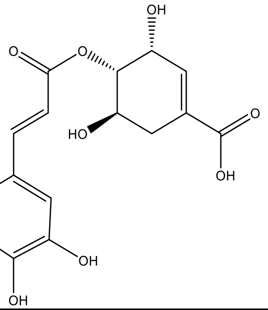	positive
caffeoyl-3-*O*-shikimic acid<br>(CAS 180981-12-8), Benchchem	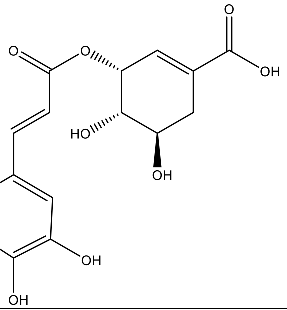	positive
4-coumaroyl-5-*O*-shikimic acid<br>synthesized enzymatically in<br>Petersen lab	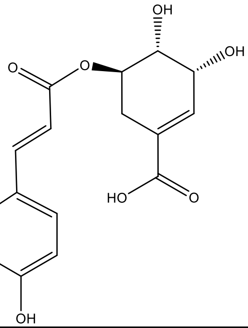	positive<br>(traces)
caffeoyl-5-*O*-quinic acid<br>= chlorogenic acid<br>(CAS 327-97-9), Carl Roth	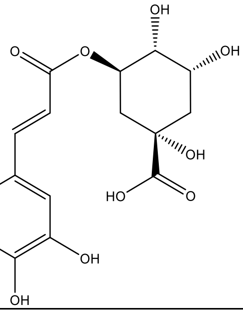	positive

caffeoyl-4-*O*-quinic acid<br>= cryptochlorogenic acid (CAS 905-99-7), Sigma Aldrich	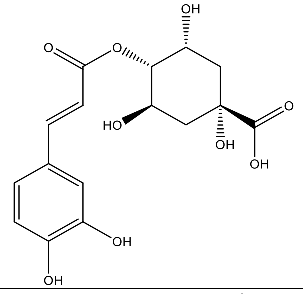	positive (traces)
caffeoyl-3-*O*-quinic acid<br>= neochlorogenic acid (CAS 180981-12-8), Phyproof	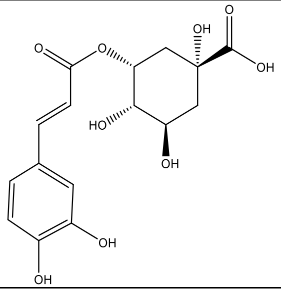	positive (traces)
N-(caffeoyl)-5-hydroxy-anthranilic acid<br>= avenanthramide A (CAS 108605-70-5), Sigma Aldrich	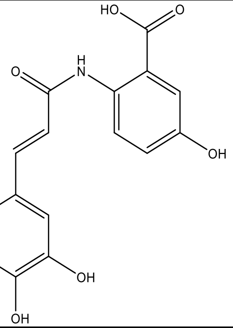	positive (traces)
4-coumaroyl-5-*O*-quinic acid (CAS 5746-55-4), Targetmol	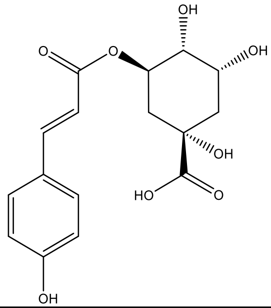	positive (traces)
4-coumaroyl-4-*O*-quinic acid (CAS 1108200-72-1), Benchchem	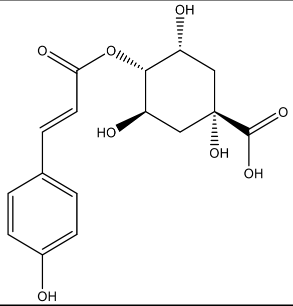	negative
4-coumaroyl-3-*O*-quinic acid (CAS 87099-71-6), Benchchem	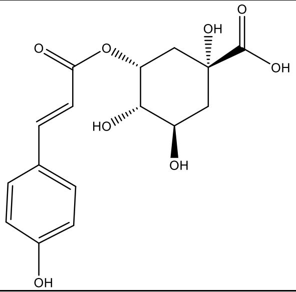	negative

rosmarinic acid<br>isolated from *Coleus blumei* suspension cultures in Petersen lab	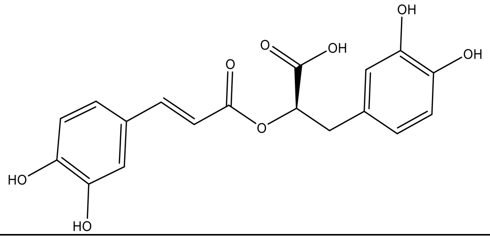	negative

Table S2 Blastp results for the query into the *Anthoceros agrestis* BONN genome (https://www.hornworts.uzh.ch) using AtCSE (At1g52760) as bait. The cutoff e-value was set at ≤ 1.0e-50

Scaffold name	Identity [%]	Alignment [aa]	e-value
Sc2ySwM_117.2740.1	53.38	166/311	2.84e-118
Sc2ySwM_117.1645.2	38.99	124/318	4.55e-77
Sc2ySwM_117.1645.3	38.99	124/318	4.55e-77
Sc2ySwM_117.1645.1	38.20	123/322	5.57e-77
Sc2ySwM_117.1304.1	40.88	130/318	1.42e-65
Sc2ySwM_362.553.1	40.07	109/272	3.01e-65
Sc2ySwM_362.551.1	37.33	109/292	1.58e-63
Sc2ySwM_362.482.1	34.97	100/286	3.69e-52

Table S3 BLASTp results for putative CSEs in the *Mesotaenium endlicherianum* genome (WDCW) in the 1kp database. AaCSE1 (GenBank: PV962689) was used as bait sequence and the cutoff e-value was set at ≤ 1.0e-50

Description	Identity [%]	e-value
WDCW_scaffold_2047059	43	3.41e-76
WDCW_scaffold_2007872	40	2.49e-73
WDCW_scaffold_2008809	40	6.98e-67
WDCW_scaffold_2009707	39	2.46e-60

Table S4 List of primers used for PCR amplifications, restriction sites are underlined

Name	Sequence (5‘-3‘)	Restriction site
AaCSE1-f-NdeI	TACATATGGCCTCCGCAGCGCCGCC	NdeI
AaCSE1-r-XhoI	TACTCGAGTCACGACTTGATCTGGCTGTCATGCTTCTCC	XhoI

MeCSE1-f-NdeI	TACATATGAAAAGTGATGTGGAACCAACTG	NdeI
MeCSE1-r-XhoI	TACTCGAGTCACGCCTGCTCGGCTG	XhoI
MeCSE2-f-NdeI	TACATATGGAGTTGCAATGGGTCTGGCT	NdeI
MeCSE2-r-XhoI	TACTCGAGTCACGCAGTGACGGTGCTGC	XhoI
MeCSE3-f-NdeI	TACATATGGCGGAAGGAAAGCGGGAC	NdeI
MeCSE3-r-XhoI	TACTCGAGTCACAGCGCAGAGGGCAG	XhoI
MeCSE1-f-NdeI	TACATATGAAAAGTGATGTGGAACCAACTG	NdeI
MeCSE1-r-XhoI	TACTCGAGTCACGCCTGCTCGGCTG	XhoI

Table S5 PCR assays and program for amplification of AaCSE1, MeputCSE1, MeputCSE2 and MeMAGL3

**Composition of PCR assays (25 µl)**			
5x GoTaq buffer	5 µl		
$MgCl_2$ (25 mM)	3 µl		
dNTPs (10 mM)	0.5 µl		
H_2O	14.4 µl		
Template (cDNA)	1 µl		
GoTaq polymerase (5 U/µl)	0.1 µl		
Primer 1 (10 or 100 µM)	0.5 µl		
Primer 2 (10 or 100 µM)	0.5 µl		
	**Denaturation**	**Primer annealing**	**Elongation**
1. cycle	94 °C; 120 s		
2.- 40. cycle	94 °C; 30 s	Primer T_m-5 °C; 60 s	70 °C; 90 s
End	Cooling to 10 °C		

Table S6 Assay compositions for measuring esterase activity of AaCSE1.

**Parameter**	**Composition**	**Conditions**
pH-optimum	Britton-Robinson buffer (Britton and Robinson 1931)<br>6.52 µg AaCSE1<br>1 mM chlorogenic acid	25 °C<br>t = 20 min<br>*n* = 3<br>measured pH values:<br>4.82, 5.24, 5.85, 6.08, 6.46, 6.60, 6.96, 7.10, 7.42, 7.63, 7.82, 8.02, 8.42
temperature optimum	0.1 M KPi buffer pH 6.5<br>9.87 µg AaCSE1<br>1 mM chlorogenic acid	t = 20 min<br>*n* = 3<br>temperature values [°C]:<br>13.0, 18.1, 23.4, 27.6, 32.7, 37.3, 42.6, 48.8, 53.3
**Variable substrate**	**Composition**	**Conditions**
caffeoyl-5-*O*-quinic acid (chlorogenic acid)	0.1 M KPi buffer pH 6.5<br>8 µg AaCSE1<br>0.125-10 mM caffeoyl-5-*O*-quinic acid (pH 7.0)	25 °C<br>t = 15 min<br>*n* = 9
caffeoyl-5-*O*-shikimic acid	0.1 M KPi buffer pH 6.5<br>3 µg AaCSE1<br>2.5-100 µM caffeoylshikimic acid (pH 7.0)	25 °C<br>t = 6 min<br>*n* = 9
substrate acceptance of AaCSE1	0.1 M KPi Buffer pH 6.9<br>20.78 µg AaCSE1<br>10-62.5 µM substrate (adjusted to pH 7.0) – see Table S1	25 °C<br>t = 60 min<br>*n* = 1

Table S7 Lipase assay composition for AaCSE1 and MeMAGL3, 4-NPB = 4-nitrophenyl butyrate

**Enzyme**	**Composition**	**Conditions**
AaCSE1	0.1 M KPi buffer pH 7.0<br>25 µg purified protein<br>0.5 % Triton X-100<br>0.0625-4 mM 4-NPB	25 °C<br>t = 15 min, only linear range was considered<br>*n* = 3
MeCSE3	0.1 M KPi buffer pH 7.0<br>15 µg purified protein<br>0.5 % Triton X-100<br>0.125-10 mM 4-NPB	25 °C<br>t = 15 min, only linear range was considered<br>*n* = 12

Table S8 Accession number and organism of sequences used in the phylogenetic tree calculation (MEGA11). The sequence marked with * has been elongated and corrected in comparison to the Uniprot sequence deduced from the translated transcript sequence (from Pp6c19_7630V6.2) since a premature stop codon has been detected

Accession Number	Species and family
AagrBONN_evm.model.Sc2ySwM_117.1304.1	*Anthoceros agrestis*, Anthocerotaceae
AagrBONN_evm.model.Sc2ySwM_117.1645.2	
PV962689 (AaCSE1)	
AagrBONN_evm.model.Sc2ySwM_362.551.1	
AagrBONN_evm.model.Sc2ySwM_362.553.1	
At1g52760_MAGL3	*Arabidopsis thaliana*, Brassicaceae
At1g11090_MAGL1	
At1g18360_MAGL2	
At1g73480_MAGL4	
At2g39400_MAGL6	
At2g39410_MAGL7	
At2g47630_MAGL9	
At3g55180_MAGL10	
At3g62860_MAGL12	
At5g14980_MAGL14	
At5g16120_MAGL15	
At5g19290_MAGL16	
Azfi_s0015.g013761	*Azolla filiculoides*, Salviniaceae
Bradi2g54367.1.p	*Brachypodium distachyon*, Poaceae
Bradi4g45350.1.p	
Cagra.0664s0023.1.p	*Coreopsis grandiflora*, Asteraceae
Cre01.g028250.t1.2	*Chlamydomonas reinhardtii*, Chlamydomonadaceae
Cre01.g038900.t1.1	
Csubellipsoidea_35947	*Coccomyxa subellipsoidea*, Coccomyxaceae
Csubellipsoidea_40270	
D8SCH9	*Selaginella moellendorffii*, Selaginellaceae
D8SGQ4	
D8T3P7	
D8TA66	
MedTru_4g127220	*Medicago truncatula*, Fabaceae
Micromonas_sp_80225	*Micromonas species*, Mamiellaceae
onekp|VITX_scaffold_2096993	*Blechum spicant* syn. *Struthiopteris spicant*, Blechnaceae
onekp|VITX_scaffold_2097046	
onekp|VITX_scaffold_2097143	
onekp|VITX_scaffold_2097379	
Osativa_LOC_Os12g16180.1	*Oryza sativa*, Poaceae
OSHQ_scaffold_2011180	*Sarcandra glabra*, Chloranthaceae
OSHQ_scaffold_2046838	
OSHQ_scaffold_2047252	
OSHQ_scaffold_2047771	
OSHQ_scaffold_2047849	
Pabies_MA_93032g0010	*Picea abies*, Pinaceae
Pabies_MA_9470692g0010	
Penium_pm008905g0060	*Penium margaritaceum*, Peniaceae
PopTri_Potri.001G17500	*Populus trichocarpa* (*P. alba* × *P. glandulosa*), Salicaceae
PopTri_Potri.003G059200	
Sacu_v1.1_s0121.g021426	*Salvinia cucullata*, Salviniaceae

scaffold-WDCW-2007872-Mesotaenium_endlicherianum PV962726_MeputCSE1	*Mesotaenium endlicherianum*, Mesotaeniaceae
scaffold-WDCW-2008809-Mesotaenium_endlicherianum PV962700_MeMAGL3	
scaffold-WDCW-2047059-Mesotaenium_endlicherianum PV962727_MeputCSE2	
SolTub_M1A3Z7	*Solanum tuberosum*, Solanaceae
TnS000078877t04	*Gnetum montanum*, Gnetaceae
Uniprot_A0AAF6BHZ7	*Marchantia polymorpha*, Marchantiaceae
Uniprot_A0A2R6W8P3	
Uniprot_A0A2R6X2N3	
Uniprot_A0A176W3K8	
Uniprot_A0A2R6XPM5	
Uniprot_A0A2K1IYG0*	*Physcomitrium patens*, Funariaceae
Uniprot_A9S0T6	
Uniprot_A9S282	
Uniprot_A9SHR3	
Uniprot_A0A2K1INR4	
Vocar.0001s0802.1.p	*Volvox carteri*, Volvocaceae

Table S9 EMBOSS Needle alignment as pairwise comparison of CSE/MAGL identified in *Anthoceros agrestis* and *Mesotaenium endlicherianum* in comparison to CSE from *Arabidopsis thaliana* (AtCSE = At1g52760). Numbers represent identity/similarity in %

	**AtCSE**	**AaCSE1**	**MeputCSE1**	**MeputCSE2**	**MeMAGL3**
**AtCSE**	100/100	51.5/68.6	36.9/56.9	30.7/45.7	30.4/46.6
**AaCSE1**		100/100	37.5/54.0	33.2/44.8	32.4/48.2
**MeputCSE1**			100/100	36.4/52.5	37.4/55.2
**MeputCSE2**				100/100	40.4/56.7
**MeMAGL3**					100/100

Table S10 EMBOSS Needle alignment as pairwise comparison of CSE and putative CSEs identified in *Anthoceros agrestis*, *Physcomitrium patens, Marchantia polymorpha* in comparison to the CSE from *Arabidopsis thaliana* (AtCSE = At1g52760). Numbers represent identity/similarity in %

	**AtCSE**	**AaCSE1**	***Pp A0A2K1IYG0**	**Mp A0AAF6BHZ7**
**AtCSE**	100/100	51.5/68.6	46.4/63.0	39.7/57.6
**AaCSE1**		100/100	46.0/63.6	43.9/60.1
**Pp A0A2K1IYG0**			100/100	38.6/57.7
**Mp A0AAF6BHZ7**				100/100

*verlängerte Pp-Sequenz wegen vorzeitigen Stoppcodons

References

Britton HTS, Robinson RA (1931) CXCVIII. – Universal buffer solutions and the dissociation constant of veronal. J Chem Soc (Resumed), 1456–1462

Cornish-Bowden A, Eisenthal R (1978) Estimation of Michaelis constant and maximum velocity from the direct linear plot. Biochim Biophys Acta 523:268–272. https://doi.org/10.1016/0005-2744(78)90030-X

de Vries S, Fürst-Jansen JMR, Irisarri I, Dhabalia Ashok A, Ischebeck T, Feussner K, Abreu IN, Petersen M, Feussner I, de Vries J (2021) The evolution of the phenylpropanoid pathway entailed pronounced radiations and divergences of enzyme families. Plant J 107:975–1002. https://doi.org/10.1111/tpj.15387

Vijayaraj P, Jashal CB, Vijayakumar A, Rani SH, Venkata Rao DK, Rajasekharan R (2012) A bifunctional enzyme that has both monoacylglycerol acyltransferase and acyl hydrolase activities. Plant Physiol 160:667–683. https://doi.org/10.1104/pp.112.202135

Wang X, Chao N, Zhang M, Jiang X, Gai Y (2019) Functional Characteristics of Caffeoyl Shikimate Esterase in *Larix kaempferi* and Monolignol Biosynthesis in Gymnosperms. Int J Mol Sci 20:6071. https://doi.org/10.3390/ijms20236071
